# The lncRNA Snhg1-Vps13D vesicle trafficking system promotes memory CD8 T cell establishment via regulating the dual effects of IL-7 signaling

**DOI:** 10.1038/s41392-021-00492-9

**Published:** 2021-03-24

**Authors:** Yanyan Zhang, Baohua Li, Qiang Bai, Pengcheng Wang, Gang Wei, Zhirong Li, Li Hu, Qin Tian, Jing Zhou, Qizhao Huang, Zhiming Wang, Shuai Yue, Jialin Wu, Liuqing Yang, Xinyuan Zhou, Lubin Jiang, Ting Ni, Lilin Ye, Yuzhang Wu

**Affiliations:** 1 Institute of Immunology PLA, Third Military Medical University, Chongqing, 400038 China; 2 Institute of Hepatopancreatobiliary Surgery, Chongqing General Hospital, University of Chinese Academy of Sciences, Chongqing, 401121 China; 3 Laboratory of Immunophysiology, GIGA Institute, Liège University, Liège, 4000 Belgium; 4 Faculty of Veterinary Medicine, Liège University, Liège, 4000 Belgium; 5 Human Phenome Institute, Fudan University, Shanghai, 200438 China; 6 Department of Molecular and Cellular Oncology, The University of Texas MD Anderson Cancer Center, Houston, 77030 TX USA; 7 Institute Pasteur of Shanghai, Chinese Academy of Sciences (CAS), Shanghai, 200031 China

**Keywords:** Adaptive immunity, Cell biology, Infection, Non-coding RNAs

## Abstract

The efficient induction and long-term persistence of pathogen-specific memory CD8 T cells are pivotal to rapidly curb the reinfection. Recent studies indicated that long-noncoding RNAs expression is highly cell- and stage-specific during T cell development and differentiation, suggesting their potential roles in T cell programs. However, the key lncRNAs playing crucial roles in memory CD8 T cell establishment remain to be clarified. Through CD8 T cell subsets profiling of lncRNAs, this study found a key lncRNA-Snhg1 with the conserved naive^hi^-effector^lo^-memory^hi^ expression pattern in CD8 T cells of both mice and human, that can promote memory formation while impeding effector CD8 in acute viral infection. Further, Snhg1 was found interacting with the conserved vesicle trafficking protein Vps13D to promote IL-7Rα membrane location specifically. With the deep mechanism probing, the results show Snhg1-Vps13D regulated IL-7 signaling with its dual effects in memory CD8 generation, which not just because of the sustaining role of STAT5-BCL-2 axis for memory survival, but more through the STAT3-TCF1-Blimp1 axis for transcriptional launch program of memory differentiation. Moreover, we performed further study with finding a similar high-low-high expression pattern of human *SNHG1/VPS13D/IL7R/TCF7* in CD8 T cell subsets from PBMC samples of the convalescent COVID-19 patients. The central role of Snhg1-Vps13D-IL-7R-TCF1 axis in memory CD8 establishment makes it a potential target for improving the vaccination effects to control the ongoing pandemic.

## Introduction

Understanding how immunological memory is established and maintained lays the foundation to improve vaccine-design. Memory CD8 T cells can survive in the human body for a long time, mediating rapid and enhanced immune response to become the key immune cells against infections and tumors. In an acute viral infection, after ~8 days with the elimination of the virus, most effector cells will die during the contraction phase (day 8 to day 30 post-infection), leaving a small pool of T cells (5–10%) to become long-lived memory cells that are poised to respond rapidly if reinfection occurs.^1^ In the course of memory CD8 T cell establishment, transcription factors and cytokines were found to play important roles in memory CD8 T cell generation and maintenance.^1,2^ Nevertheless, how those cytokine signals integrate with transcription factors to induce memory formation and balance effector-memory progress remains to be clarified. For instance, it was thought IL-7 mainly activates BCL-2 through STAT5 for cell survival during memory generation in the previous study.^3^ Whether IL-7 can directly promote memory differentiation through transcription factors remain unclear. What’s more, the transcription factor TCF-1 was found to play a key role in memory CD8 T cell differentiation from mouse to human.^4–6^ Moreover, TCF-1 was thought to be induced by canonical Wnt-β-Catenin signaling in memory CD8 generation. However, β-Catenin knockout did not injure memory CD8 T cell formation,^7^ and p45 was dispensable for generating cytotoxic/effector CD8 T cells or maintaining the memory CD8 T cell pool (two isoforms of TCF-1, the long isoform p45 containing a unique N-terminal β-Catenin–interacting domain compared with the short isoform p33).^5,6^ Moreover, p33 isoforms lacking the β-Catenin binding domain can effectively hinder effector CD8 T cell formation.^8^ As a result, whether TCF-1 is induced by canonical Wnt-β-Catenin signaling in memory CD8 generation remains controversial. And the mechanism of how TCF-1 promotes memory with blocking effector remains to be elucidated.

Recent studies have shown that lncRNAs expression is highly cell- and stage-specific during T cell development and differentiation, suggesting their potential roles in T cell programs.^9^ While only ~2% of the human genome are found as protein-coding regions (~20,000 genes), a major part of the genome (more than 80%) give rise to noncoding RNAs (ncRNAs). Although many short ncRNAs, such as microRNAs (miRNAs), are widely studied, the regulatory functions of a variety of lncRNAs with a length longer than 200 nucleotides are largely unknown. The version 19 release from GenCode has annotated 14,000 lncRNA genes in humans, showing that lncRNAs are expressed in a disease-, tissue- or developmental stage-specific manner. The data indicated that ~50–60% of lncRNAs were cell-stage- or cell-subset-specific, in contrast to 6–8% of mRNAs. Meanwhile, up to 80% of protein-coding genes were shared by subsets within a group, in contrast to just ~15% of the lncRNAs. Approximately 56% of lncRNAs exhibited preferential expression in one T cell subset compared to the others, suggesting their potential roles in T cell programs.^10–12^

Although various lncRNAs have been elucidated to play a role in oncology, with some of them demonstrated involving in innate immunity, the key lncRNAs regulating CD8 T cell differentiation remain unclear. Here, using lncRNA-seq of CD8 T cell subsets, we found a key lncRNA-Snhg1 (Small nucleolar RNA host gene 1) with the conserved high-low-high expression pattern in naive-effector-memory CD8 T cells from mouse to human, was required for memory CD8 T cells especially Tcm (central memory) differentiation. Snhg1 pulldown coupled MS (mass spectrometry) identified its interacting protein, the conserved membrane trafficking protein-Vps13D (Vacuolar protein sorting 13 homolog D). Further, we found that both retrovirus-mediated Snhg1 and Vps13D depletion restricted IL-7Rα (CD127) trafficking from ER-Golgi to cell membrane specifically, which impaired memory CD8 T cell differentiation through the previously ill-defined IL-7-STAT3-TCF1-Blimp1 axis.

## Results

### LncRNA Snhg1 exhibiting the naive^hi^-effector^lo^-memory^hi^ expression pattern in T cell subsets from mouse to human is required for memory CD8 T cell establishment

**Fig. 1:**
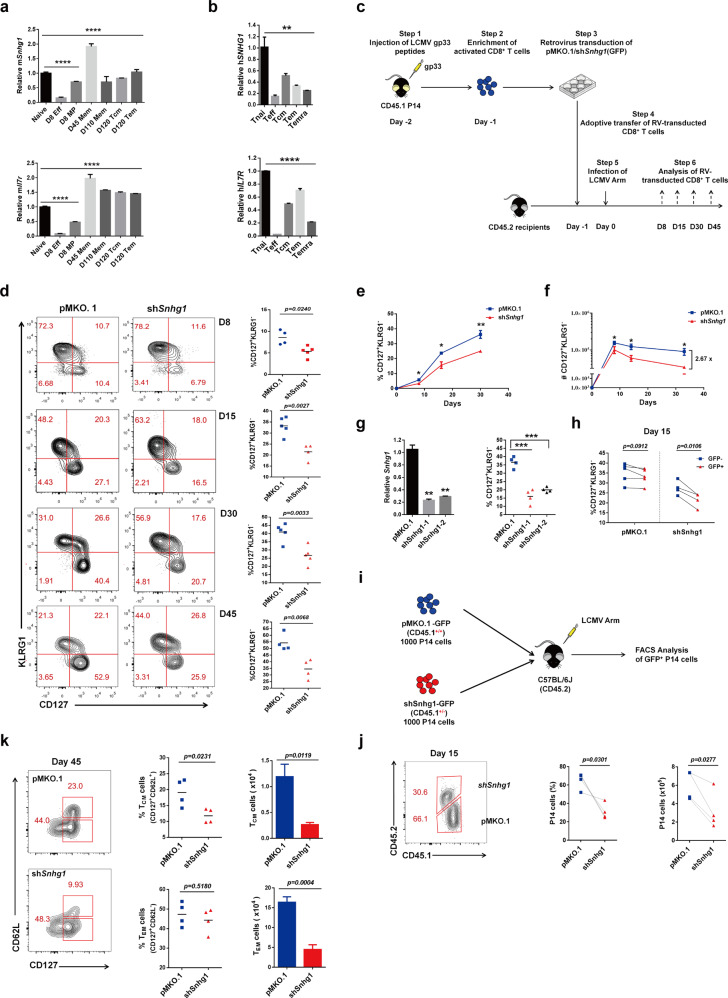
LncRNA Snhg1 exhibiting naive^hi^-effector^lo^-memory^hi^ expression pattern in T cell subsets from mouse to human is required for memory CD8 T cell establishment. **a**, **b** m*Snhg1/*h*SNHG1* and m*Il7r*/h*IL7R* transcripts level were validated by RT-qPCR in the indicated CD8 T cell lineages from LCMV-infected mice (a) or human peripheral blood mononuclear cells (PBMCs) (**b**). **c** The sketch of retroviral transduction experiments of P14 CD8^+^ T cells in C57BL/6J mice. **d** Flow cytometry with quantification of CD127^+^KLRG1^−^ population in GFP^+^ P14 CD8^+^ T cells on indicated days p.i. (post infection). **e**–**f** 2000 GFP^+^ P14 cells (CD45.1) transducted with retrovirus expressing pMKO.1 or Snhg1 shRNA were sorted and injected into CD45.2 mice following LCMV infection, the cell proportion and cell number kinetics of CD127^+^KLRG1^−^ GFP^+^ P14 cells were shown in (**e**) and (**f**), respectively. **g** The knockdown efficiency of the two shRNA sequences of *Snhg1* in sorted memory CD8 T cells was shown with RT-qPCR analysis (left), with the FACS quantification of the two shRNAs of Snhg1 to memory CD8 T cell formation compared with pMKO.1 on day 30 p.i. (right). **h** The internal analysis of CD127^+^KLRG1^−^ population in GFP^−^ and GFP^+^ P14 CD8 T cells in the same mice from pMKO.1 or sh*Snhg1* groups on day 15 p.i. **i**, **j** 1000 CD45.1^+/+^ pMKO.1 P14 and 1000 CD45.1^+/−^ sh*Snhg1* P14 were sorted and co-transferred into CD45.2 mice following with LCMV infection as indicated (**i**). Flow cytometry analysis with both cell proportion and cell number of pMKO.1 or sh*Snhg1* P14 cells in total P14 cells on day 15 p.i. (**j**). **k** The sorted 2000 GFP^+^ P14 cells (CD45.1) transducted with retrovirus expressing pMKO.1 or sh*Snhg1* were injected into CD45.2 mice following with LCMV infection, the frequency and number of Tcm and Tem cells in pMKO.1 or sh*Snhg1* P14 CD8^+^ T cells were analyzed on day 45 p.i. Data are representative of two or three independent experiments with at least three replicates or four mice per group (error bars denote s.e.m.). *p < 0.05, **p < 0.01, ***p < 0.001, ****p < 0.0001 (paired or unpaired two-tailed *t*-test). See also Fig. S1, Table S1–3

First of all, to identify key lncRNAs in memory CD8 T cell differentiation, we sorted naive (CD44^−^CD62L^+^), effector (day 8 post-infection with CD44^+^CD62L^−^KLRG1^+^) and memory CD8 T cells (day 30 p.i. with CD44^+^CD127^+^) from the LCMV Armstrong infected mouse for lncRNA-seq analysis (Supplementary Fig. S1a, Supplementary Table S1).^13^ Thus these lncRNAs were classified based on expression level to the naive-up, memory-up, effector-up, and naive-effector-memory high-low-high groups (Supplementary Fig. S1b). The genes with the naive-effector-memory high-low-high expression pattern usually play important roles in memory CD8 T cell differentiation, such as *Il7r*, *Sell, Tcf7, Cd27*, and *Ccr7*, which shut down their expression when naive T cells encounter antigen to be activated as effectors and resume once the pathogen has been cleared and the resting memory T cells form.^14,15^ From this group of lncRNAs, we tested the top candidates (Supplementary Fig. S1c, Supplementary Table S2) and identified the lncRNA Snhg1, that with a typical high-low-high expression pattern in naive-effector-memory CD8 T cells, which is similar to that of *Il7r* (Supplementary Fig. 1a, Supplementary Table S3). Both of them showed high expression in all memory lineages, including day 8 memory precursors (MP, KLRG1^−^CD127^+^), day 45 memory cells (KLRG1^−^CD127^+^), day 110 memory cells, day 120 central memory cells (CD127^+^CD62L^+^) and effector memory cells (CD127^+^CD62L^−^). Since mouse *Snhg1* shows a certain degree of conservation compared to human *SNHG1*, we detected the h*SNHG1* expression pattern in naive (CD45RA^+^CCR7^+^), effector (induced naive hCD8 T cells using anti-hCD3/anti-hCD28 for 3 days in vitro culturing with IL-2), central memory (Tcm, CD45RA^−^CCR7^+^), effector memory (Tem, CD45RA^−^CCR7^−^) and the Temra (CD45RA^+^CCR7^−^) CD8 T cells from human PBMCs (Supplementary Table S1), with *IL7R* gene tested together as control. Human CD8 T cells can be divided into at least four different subsets based on their phenotype and functions: naive and central memory (Tcm) T cells display high proliferative potential and lack of an immediate effector functions whereas effector memory (Tem) and CD45RA^+^ effector memory (Temra) T cells have low proliferative capacities but produce cytokines and exert cytotoxic activity, respectively. CD45RA^+^ effector Temra cells represent the most differentiated type of memory cells; they have a high susceptibility to apoptosis and express high levels of cytotoxic molecules such as perforin and Fas ligand.^16^ And the h*SNHG1* showed a similar high-low-high expression pattern as that of m*Snhg1* (Fig. 1b, Supplementary Table S3). As contrast to lncRNAs, we investigated mRNAs in naive, effector, and memory CD8 T cells in the meantime, and found lncRNAs holistically had lower expression than mRNAs but showed more specific regulation (Supplementary Fig. S1d). We likewise classified the mRNAs based on their expression levels to naive-up, memory-up, effector-up, and naive-effector-memory high-low-high groups (Supplementary Fig. S1e). As expected, the effector-up genes, such as *Granzyme* gene isoforms, were shown in the naive-effector-memory low-high-low group; while the memory-up genes, such as *Il7r*, were in the naive-effector-memory high-low-high group, indicating the RNA-seq worked well (Supplementary Fig. S1e).

The lncRNA Snhg1 is an integrated form of the 9 exons of the gene *Snhg1*, the introns of which are for housekeeping snoRNAs that play key roles in regulating mRNA splicing in the nucleus, as indicated by its full name, “Small nucleolar RNA host gene 1”.^17^ This phenomenon has largely limited the ability to obtain *Snhg1* gene knockout mice. Only a few reports about human SNHG1 focusing on its oncogenic role in tumors have been published, whereas little is known about its role in immunology. To investigate the role of lncRNA Snhg1 in memory CD8 T cells, we applied the pMKO.1-shRNA retrovirus transduction system to integrate shRNA of *Snhg1* into the genome of virus-specific P14 cells, which express a transgenic TCR specifically recognizing LCMV glycoprotein gp33–41 epitope (H2-D^b^) (Fig. 1c, Supplementary Table S3). We transferred pMKO.1 (IRES-GFP) control or sh*Snhg1* transducted P14 cells (CD45.1) to C57BL/6 J recipient mice (CD45.2) that were subsequently subjected to LCMV-Armstrong infection. With FACS analysis on days 8, 15, 30, and 45 post-infection, we found that lncRNA Snhg1 depletion decreased memory CD8 T cell (CD127^+^KLRG1^−^) formation both in the expansion (day 8) and contraction (days 15, 30) phases (Fig. 1d). We next sorted and injected the same quantity of 2000 GFP^+^ P14 cells that transducted with pMKO.1 or sh*Snhg1* into mice, respectively, then we analyzed both cell proportion and cell number kinetics of CD127^+^KLRG1^−^ P14 CD8 T cells along timeline of post-infection, and found that Snhg1 affected memory CD8 T cell generation both in the expansion (day 8) and contraction (days 15, 30) phases (Fig. 1e, f). To further confirm the non-off-target effects of Snhg1, we tested the other shRNA sequence of Snhg1 (sh*Snhg1*-2) (Supplementary Table S3), which showed similar results on day 30 post-infection (Fig. 1g); and the knockdown efficiency of both the shRNA sequences were verified as well (Fig. 1g). As a contrast to the above external assay between mice, for the internal analysis we obtained similar results of impaired memory CD8 T cells by comparing the GFP^−^ with GFP^+^ P14 CD8 T cells in the same mice in control or sh*Snhg1* groups on day 30 post-infection, respectively (Fig. 1h). To double-confirm the impairment of Snhg1 knockdown to memory formation, we sorted 1000 CD45.1^+/+^ pMKO.1-GFP^+^ P14 cells with 1000 CD45.1^+/−^ shSnhg1-GFP^+^ P14 cells and co-transferred them into the same mouse as indicated in Fig. 1i, and analyzed cell proportion with cell number. The results show that Snhg1 depletion obviously impairs memory P14 T cells on day 15 post-infection (Fig. 1j). This result of co-transfer experiment is consistent with both results of the external and internal analysis as showing above. Depletion of Snhg1 also impaired both cell proportion and cell number of central memory T cells (Tcm, CD127^+^CD62L^+^) and cell number of effector memory T cells (Tem, CD127^+^CD62L^−^) on day 45 (Fig. 1k). For the candidates screening experiments, we first applied RT-qPCR validation for their expression pattern, then we designed the shRNAs targeted the candidates to measure the Log2 ratio of CD127^+^KLRG1^−^ memory CD8 frequency in GFP^−^ to that of GFP^+^ P14 cells for internal analysis on days 8, 15, 30, respectively, with finding that the top candidate Snhg1 (NONMMUG020761) plays a major role in memory CD8 differentiation, while the other candidates did not show the appreciable effects on memory formation as that of Snhg1 (Supplementary Fig. S1i). Finally, we show the gating strategy for the above FACS analysis in supplementary Fig. 1. Supplementary Fig. S1f is the gating strategy for Fig. 1d, g, h; Supplementary Fig. S1g is for Fig. 1e, f, k; and Supplementary Fig. S1h is for Fig. 1j. In brief, these data suggested lncRNA Snhg1 is required for memory CD8 T cells especially Tcm differentiation.

### The vesicle trafficking protein Vps13D interacted with Snhg1 from mouse to human is essential for memory CD8 T cell differentiation

**Fig. 2:**
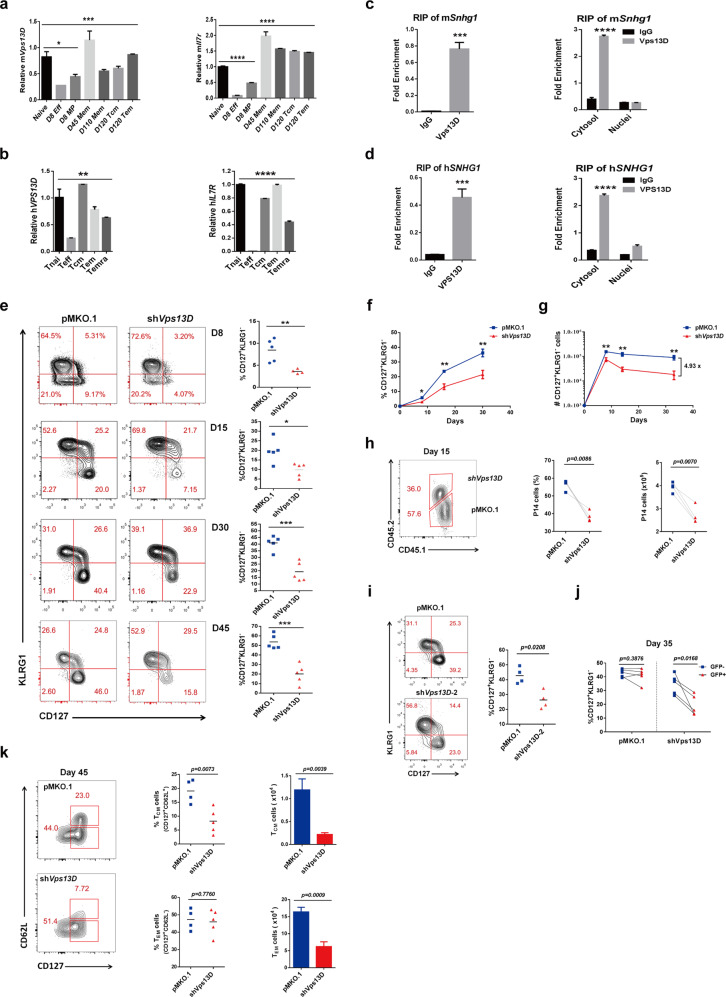
The vesicle trafficking protein Vps13D interacts with Snhg1 from mouse to human is essential for memory CD8 T cell differentiation. **a**, **b** RT-qPCR analysis of m*Vps13D* (h*VPS13D*) or m*Il7r* (h*IL7R*) in different CD8 T cell lineages of the mouse (or human) (experiments done side by side with that of Fig. 1a, b). **c**, **d** RIP assay of m*Snhg1*/h*SNHG1*) with m*Vps13D*/h*VPS13D* in EL4 (**c**) or Jurkat (**d**) cells with in nuclear-cytoplasmic fractionation experiments (right), respectively. **e** Flow cytometry analysis of CD127^+^KLRG1^−^ population in control or sh*Vps13D* GFP^+^ P14 CD8 T cells on days 8, 15, 30, 45 p.i. (D30 experiments done side by side with that of Fig. 1d). **f**, **g** The cell proportion (**f**) and cell number (**g**) kinetics of CD127^+^KLRG1^−^ GFP^+^ P14 cells with pMKO.1/shVps13D (experiments done side by side with that of Fig. 1e, f). **h** Flow cytometry with cell proportion and cell number of pMKO.1/sh*Vps13D* P14 cells in total P14 cells on day 15 p.i. **i** Flow cytometry analysis of CD127^+^KLRG1^−^ cells in GFP^+^ P14 (CD45.1) cells transducted with retrovirus expressing empty vector or sh*Vps13D*-2 on day 35 p.i. **j** The internal analysis of CD127^+^KLRG1^−^ population in GFP^−^ and GFP^+^ P14 CD8 T cells in the same mice in control or sh*Vps13D* groups on day 35 p.i. **k** Flow cytometry with cell proportion and cell number of Tcm and Tem in control or sh*Vps13D* P14 CD8^+^ T cells on day 45 p.i. (experiments done side by side with that of Fig. 1k). Data are representative of two or three independent experiments with at least three replicates or four mice per group (error bars denote s.e.m.). *p < 0.05, **p < 0.01, ***p < 0.001, ****p < 0.0001 (paired or unpaired two-tailed *t*-test). See also Fig. S2, Tables S1, 3–4

To uncover the proteins that interact with Snhg1 for mechanistic probing, we sorted memory CD8 T cells for RNA pulldown-coupled mass spectrometry analysis using the antisense of Snhg1 as the control for sense of Snhg1 pulldown analysis. The enriched hits from the mass spectrum showed three proteins as indicated (Supplementary Fig. S2a, Supplementary Table S4). The enriched proteins shown with Vps13D are the overlapped ones of two independent experiments that have excluded the false positive hits from the antisense control. We focused on Vps13D that mainly associates with membrane trafficking,^18,19^ as we found gene *Vps13D* has the typical high-low-high expression pattern in naive-effector-memory CD8 T cells (Fig. 2a). As gene *Vps13D* is highly conserved from mouse to human, we further detected the expression pattern of human *VPS13D* in naive, effector, and memory CD8 T cells with *IL7R* detected together as control (Supplementary Table S3). We found that *Vps13D* has a similar high-low-high expression pattern to that of *Snhg1* both in mouse and human naive, effector, and memory T cells (Fig. 2a, b), suggesting their latent roles in memory CD8 T cell establishment. To confirm the interaction between Snhg1 and Vps13D, we further applied RIP (RNA immunoprecipitation) assay in a mouse T cell line-EL4 cells and a human T cell line-Jurkat cells, respectively, with demonstrating the binding of mSnhg1/hSNHG1 and mVps13D/hVPS13D from mouse to human and mainly in the cytoplasm (Fig. 2c, d, Supplementary Table S3). For RIP assay with cell fractionation, we tested m*Gapdh/*h*GAPDH* expression level and verified the cell fractionation efficiency both in EL4 and Jurkat cells (Supplementary Fig. S2b).

The particular role of Vps13D remains unclear, with only a few references reported that it plays some role in mitochondrial associated autophagy and localizes to membrane contact sites.^18–21^ Here, we assessed its function in memory CD8 T cells using the pMKO.1-shRNA retrovirus transduction system to integrate sh*Vps13D* into the genome of P14 cells (CD45.1) following adoptive transfer to recipient mice (CD45.2) (Supplementary Table S3). With FACS analysis on days 8, 15, 30, and 45 post LCMV infection, we also found that Vps13D depletion affected memory CD8 T cell precursors (CD127^+^KLRG1^−^) formation both in the expansion (day 8) and contraction (days 15, 30) phases (Fig. 2e), similar to that of Snhg1. We next analyzed the sorted and injected 2000 GFP^+^ P14 cells with both cell proportion and cell number kinetics of CD127^+^KLRG1^−^ CD8 T cells along timeline of post-infection, and found that Vps13D affected memory CD8 T cell generation both in the expansion (day 8) and contraction (day 15, 30) phases (Fig. 2f, g). To further ascertain the non-off-target effects of Vps13D, we examined another shRNA sequence of Vps13D, sh*Vps13D*-2 (Supplementary Table S3), which showed the same results on day 30 post-infection (Fig. 2i). We also confirmed the knockdown efficiency of the two shRNA sequences at both mRNA level and protein level (Fig. S2c). As a contrast to the above external analysis between mice, for the internal analysis we obtained similar results of impaired memory and Tcm cells by comparing the GFP^−^ with GFP^+^ P14 CD8 T cells in the same mice in the control or sh*Vps13D* groups on days 8, 30 and 40 post-infection (Fig. 2j; Supplementary Fig. S2d, e). To double-confirm the impairment of Vps13D knockdown to memory formation, we sorted 1000 CD45.1^+/+^ pMKO.1-GFP^+^ P14 cells with 1000 CD45.1^+/−^ shVps13D-GFP^+^ P14 cells and co-transferred them into the same mouse as indicated in Fig. 1i, and analyzed cell proportion with cell number. The results show that Vps13D depletion obviously impairs memory P14 T cells on day 15 post-infection (Fig. 2h). This result of co-transfer experiment is consistent with both results of the external and internal analysis as showing above. Depletion of Vps13D also impaired both cell proportion and cell number of central memory T cells (Tcm, CD127^+^CD62L^+^) and cell number of effector memory T cells (Tem, CD127^+^CD62L^−^) on day 45 (Fig. 2k). In short, those data indicated the essential role of Vps13D that interacting with Snhg1 in memory CD8 T cells especially Tcm differentiation.

### Snhg1 and Vps13D promote memory CD8 with impeding effectors and preserve the function of memory CD8 T cells

**Fig. 3:**
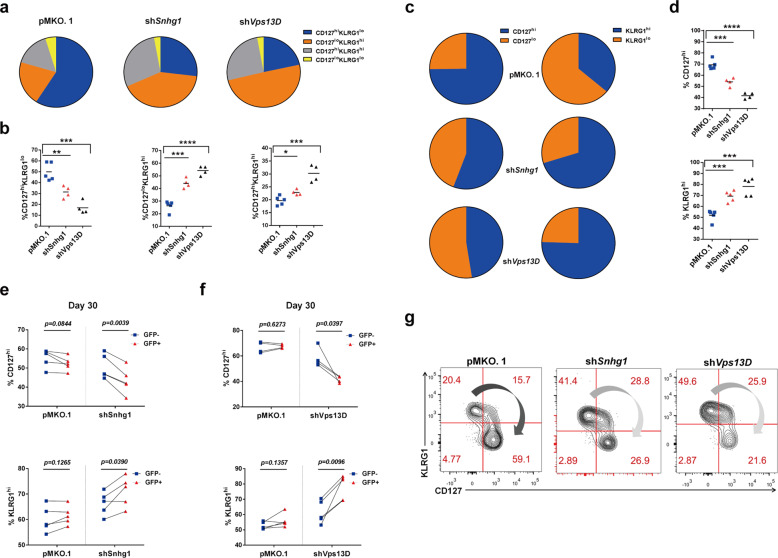
Snhg1 and Vps13D depletion impair memory while accumulate effector CD8 T cells. **a**–**d** Representative pie charts (**a**, **c**) with quantification (**b**, **d**) of the indicated cell proportion in pMKO.1/shSnhg1/shVps13D GFP^+^ P14 cells on day 45 p.i. **e**, **f** The internal analysis of indicated cell proportion by comparing the GFP^−^ with GFP^+^ P14 CD8 T cells in the same mice in control or sh*Vps13D*/sh*Snhg1* groups on day 30 p.i. **g** Representative flow cytometry to show the effect of sh*Snhg1* and sh*Vps13D* reducing memory with retention of effector T cells on day 45 p.i. Data are representative of two or three independent experiments with at least four mice per group (error bars denote s.e.m.). *p < 0.05, **p < 0.01, ***p < 0.001, ****p < 0.0001 (paired or unpaired two-tailed *t*-test). See also Fig. S3, Tables S1, 3

Furthermore, we investigated how Snhg1 and Vps13D affect memory CD8 T cell generation. To this end, we mapped and quantified the FACS data with five groups: CD127^+^KLRG1^−^, CD127^−^KLRG1^+^, CD127^+^KLRG1^+^, CD127^+^ and KLRG1^+^ alone (Fig. 3a–d). The results showed Snhg1/Vps13D depletion impaired CD127^+^KLRG1^−^ cells with accumulated CD127^−^KLRG1^+^ and CD127^+^KLRG1^+^ cells; and impaired total CD127^+^ cells with accumulated total KLRG1^+^ cells, both externally and internally (Fig. 3e, f), suggesting Snhg1 and Vps13D depletion impair memory while accumulate effector CD8 T cells (Fig. 3g). Briefly, the above data revealed that Snhg1 and Vps13D depletion impair memory CD8 with retention of effector T cells. Meanwhile, to test whether memory T cell function was impaired, we performed recall analysis on day 5 post re-infection of the sorted memory CD8 T cells as indicated in Supplementary Fig. S3a. The results showed that Snhg1 and Vps13D depletion injured memory CD8 T cell function to a certain extent, with both cell proportion and MFI of IFNγ decreased (Supplementary Fig. S3b), and impaired secondary expansion of P14 CD8 T cells (Supplementary Fig. S3c). To confirm the above results were not influenced by different viral titers, we tested the viral titers with the timeline of post-infection, and found the virus had been successfully cleared on day 8 in LCMV Armstrong acute infection (Supplementary Fig. S3d). The LCMV viral loads in the spleen were quantified by the RT-qPCR method as described in the paper from Shane Crotty’s group.^22^ In this method paper, they have shown the QPCR technique was ~1000-fold more sensitive than the conventional plaque assay, and they were able to detect a very low level of the virus by QPCR when it is not detectable by conventional plaque assay. As a result, the LCMV Armstrong is still detected at extremely low levels at late timepoints should be the issue of the detection method. In summary of the above data, Snhg1 and Vps13D promote memory with impeding effector CD8 and preserve the function of memory CD8 T cells.

### Genes modulated by Snhg1 and Vps13D in T cells mainly involve in membrane receptor-associated immune processes

**Fig. 4:**
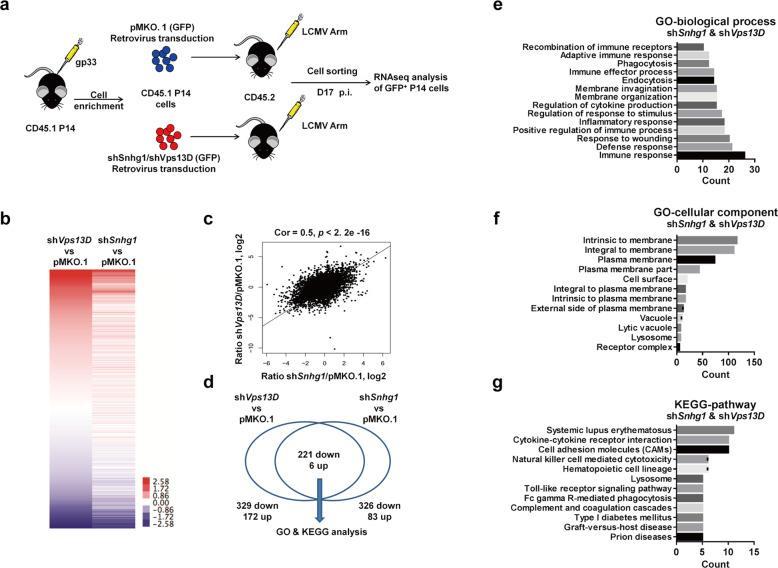
Genes modulated by Snhg1 and Vps13D in T cells mainly involve in membrane receptor-associated immune processes. **a** The gp33 activated P14 CD8 T cells from CD45.1 mice were enriched and transducted with a retrovirus containing sh*Snhg1/shVps13D* or control vector, thus adoptively transferred into CD45.2 mice and the GFP^+^ P14 CD8 T cells were sorted for RNA-seq analysis on day 17 post-infection. **b** Heatmap of the genes regulated by sh*Snhg1* or sh*Vps13D* compared with the control pMKO.1 group. **c** Correlation analysis between normalized genes expression ratio of sh*Snhg1*/pMKO.1 and sh*Vps13D*/pMKO.1 with Pearson’s correlation coefficient. **d** Venny diagram of shared upregulated or downregulated genes in sh*Snhg1* and sh*Vps13D* group. **e**–**g** GO and KEGG analysis of shared downregulated genes between sh*Snhg1* and sh*Vps13D* group in biological processes, cellular components and pathways. Data are obtained from one experiment with two biological replicates pooled from at least four mice per group. See also Fig. S4, Tables S1, 3, 5

For the mechanistic study, we first utilized RNA-seq to identify the downstream signaling modulated by Snhg1/Vps13D through sorting the indicated memory precursor T cells on day 17 post-infection in the transition stage (Fig. 4a). The following bioinformatics analysis demonstrated that genes regulated by Snhg1 and Vps13D displayed high similarity with high relevance (Fig. 4b, c). Then, we extracted the two-thirds of downregulated genes (221 genes with pre-exclusion of false positives by the control) shared by sh*Snhg1* and sh*Vps13D* for GO and KEGG analysis (Fig. 4d, Supplementary Table S5). The GO-biological process and KEGG-pathway analysis both showed that Snhg1 and Vps13D-regulated genes are mainly involved in immune responses, especially immune receptor-related progress (Fig. 4e, g). In addition, GO-cellular component analysis revealed that the immune process predominantly occurred on the cell membrane (Fig. 4f). In the meantime, analysis of genes regulated by sh*Snhg1* (326 downregulated genes with pre-exclusion of false positives by the control, Supplementary Fig. S4a–c) or sh*Vps13D* (329 downregulated genes with pre-exclusion of false positives by the control, Supplementary Fig. S4d–f) alone exhibited similar results. In brief, the above results showed that the genes modulated by Snhg1/Vps13D in T cells are mainly involved in membrane receptor-associated immune processes.

### The trafficking of IL-7Rα from ER-Golgi to cell membrane specifically depends on Snhg1 and Vps13D

**Fig. 5:**
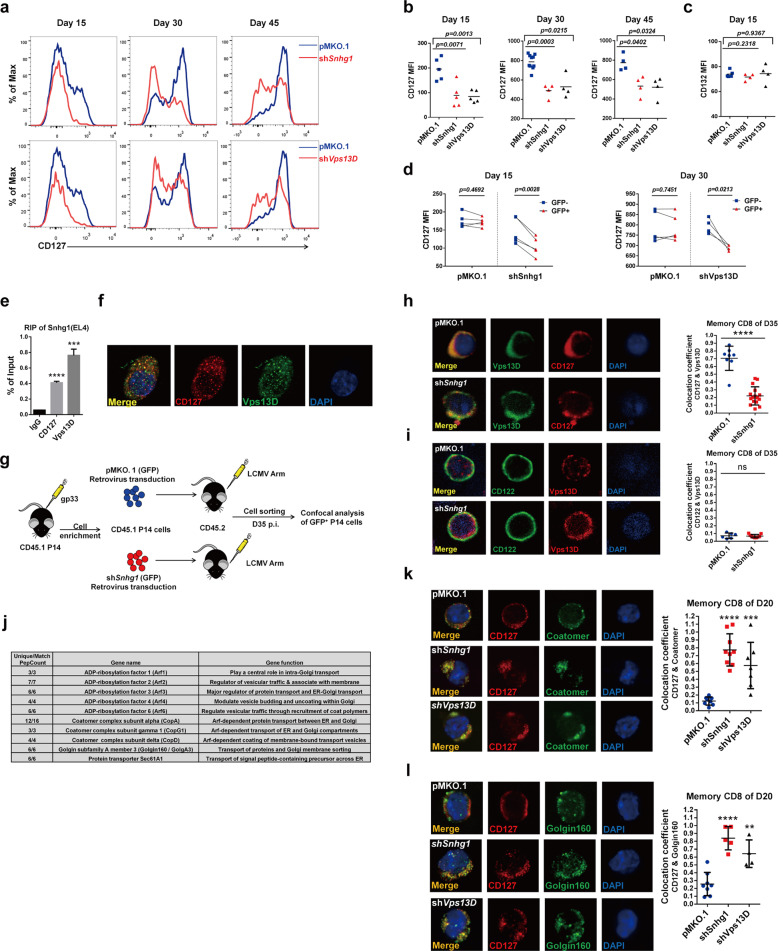
Snhg1 and Vps13D deficiency specifically block IL-7Rα trafficking from ER-Golgi to cell membrane. **a**–**c** Expression (**a**) and quantification (**b**) of CD127 in pMKO.1/sh*Snhg1*/sh*Vps13D* samples with comparison of CD132 (**c**) on indicated days p.i. **d** The internal analysis of CD127 MFI by comparing the GFP^−^ with GFP^+^ P14 CD8 T cells in the same mice in control or sh*Snhg1*/sh*Vps13D* groups on indicated days p.i. **e** RIP assay of Snhg1 using Rabbit anti-CD127 or anti-Vps13D in El4 cells compared with the normal Rabbit IgG control. **f** Confocal microscopy of CD127 (red) with Vps13D (green) in El4 cells. **g**–**i** The GFP^+^ P14 CD8 T cells were sorted as indicated (**g**) for confocal microscopy and image J quantification of the collocation coefficient of CD127 (**h**) or CD122 (**i**) with Vps13D on day 35 p.i.(the collocation coefficient means the ratio of colocalization MFI to the cell total MFI, which is not affected by protein expression level). **j** The enriched protein hits from Immunoprecipitation (IP)-based pulldown mass spectrometry of anti-Vps13D in El4 cells compared with the normal IgG control. **k**, **l** Confocal microscopy and image J quantification of the collocation coefficient of CD127 (red) with Coatomer or Golgin160 (green) in the sorted GFP^+^ P14 CD8 T cells expressing empty vector or Snhg1/Vps13D shRNA on day 20 p.i. Data are representative of two or three independent experiments with at least three replicates or four mice per group (error bars denote s.e.m.). ns not significant; **p < 0.01, ***p < 0.001, ****p < 0.0001 (paired or unpaired two-tailed *t*-test). See also Fig. S5, Tables S1, 3, 6

The cytokines IL-7 and IL-15 were found to play important roles in memory CD8 T cell generation and maintenance both in mouse and human.^23,24^ In addition, memory T cell generation is particularly IL-7-dependent,^25,26^ while the maintenance of the long-term memory pool predominantly depends on IL-15 subsequently.^27–29^ The IL-7 receptor is composed of the common gamma-chain CD132 (shared by IL-2, IL-4, IL-9, IL-15, and IL-21 receptors) and the specific IL-7 receptor alpha-chain CD127. The signals that regulate both the expression and function of IL-7R on T cells have yet to be well characterized. Since our results above suggested that Snhg1 and Vps13D involved processes in memory CD8 T cells mainly associated with membrane receptor-related immune responses, we first assessed the membrane expression intensity of CD127/IL-7Rα following Snhg1/Vps13D deficiency on days 15, 30, and 45 post-infection, with the common gamma-chain CD132 detected for comparison. The results showed that the MFI of membrane IL-7Rα was reduced through day 15 to day 45 both externally and internally, with the common gamma-chain CD132 not affected (Fig. 5a–d). To further elucidate the interaction and regulation among Snhg1, Vps13D and IL-7Rα, we first applied RIP and colocalization analysis in EL4 cells (We first detected and found there are IL-7Rα expression in mouse EL4 cells, but not in human Jurkat cells, Supplementary Fig. S5a, b). We found that both Vps13D and IL-7Rα can pulldown Snhg1 in RIP assay (Fig. 5e). To eliminate the possibility that CD127 and VPS13D may just have some affinity for RNA, we performed additional RIP experiments as showing in Fig. S5g. CD127 did not show an additional affinity for RNA of m*Gapdh* in EL4 cells, while VPS13D seems to have some affinity for RNA of h*ACTIN* in Jurkat cells, which indicated that the binding of Snhg1 is more specific for CD127 compared with Vps13D. Further on, we found IL-7Rα had colocalization with Vps13D in EL4 cells (Fig. 5f, Supplementary Fig. S5b). Next, to determine if Snhg1 plays a guiding role in recruiting its target IL-7Rα to the Vps13D vesicle trafficking system, we established mouse experiments with sh*Snhg1* (IRES-GFP) retrovirus transduction through adoptive transfer and analyzed the sorted GFP^+^ P14 CD8 T cells using confocal IF (immunofluorescence) analysis on day 35 post-infection (Fig. 5g). The results showed that Snhg1 depletion obviously decreased the colocalization between IL-7Rα and Vps13D in memory CD8 T cells (Fig. 5h, Supplementary Fig. S5c). As a contrast, CD122/IL-2Rβ (the IL-15 receptor beta-chain shared with IL-2 receptor) did not show any colocalization with Vps13D regardless of Snhg1 presence or not (Fig. 5i, Supplementary Fig. S5d).

To further elucidate the mechanism through which Snhg1 and Vps13D affect IL-7Rα membrane location, we applied IP (immunoprecipitation) coupled MS (mass spectrometry) analysis (with pre-exclusion of false positives for the rabbit anti-Vps13D sample using the rabbit normal IgG control), and found the proteins enriched in the Vps13D pulldown sample exhibited isoforms of ARF (ADP-ribosylation factor) and subunits of the Coatomer complex, with all of them play key roles in the ARF-dependent protein transport between ER (endoplasmic reticulum) and Golgi (Fig. 5j, Supplementary Table S6). To verify if Snhg1 and Vps13D play a role in IL-7Rα trafficking between ER and Golgi, we set up mouse experiments with Snhg1/Vps13D depletion and analyzed the sorted GFP^+^ P14 CD8 T cells using confocal IF analysis on day 20 (post-infection) in the transition stage. The results showed that IL-7Rα accumulated with Coatomer or Golgin160 in the ER-Golgi transport system when Snhg1 or Vps13D was depleted (Fig. 5k, l, Supplementary Fig. S5e, f). Taken together, the above results indicated that Snhg1/Vps13D depletion blocked CD127 trafficking from ER-Golgi to cell membrane specifically.

### Snhg1/Vps13D depletion impairs transcriptional launch program of memory CD8 T cells through IL-7-STAT3-TCF1-Blimp1 axis in the expansion and contraction phases

**Fig. 6:**
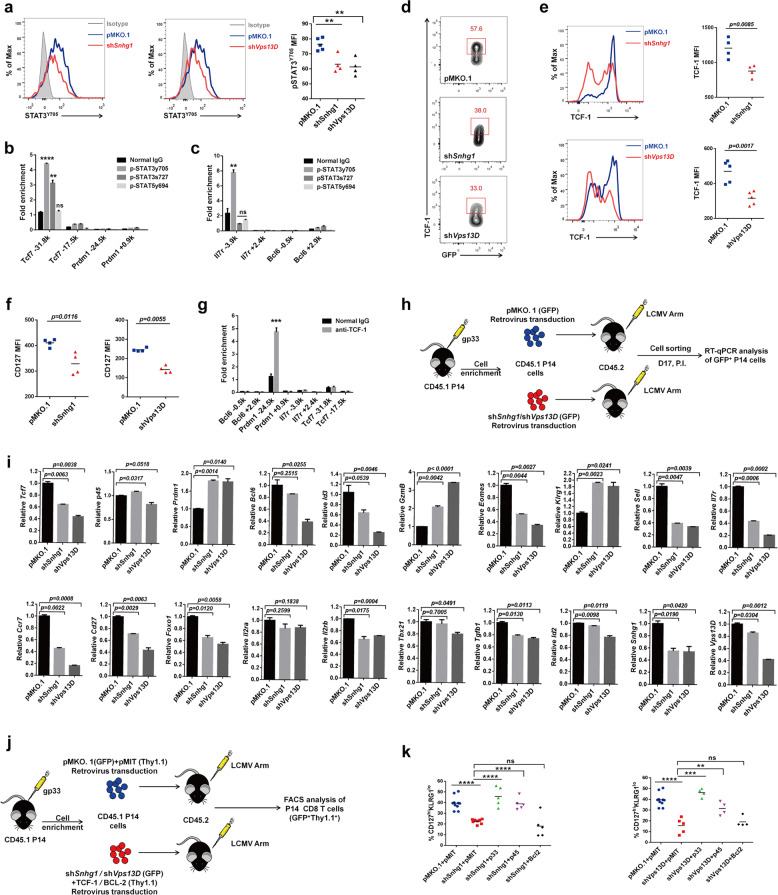
Snhg1/Vps13D depletion impairs transcriptional launch program of memory CD8 T cells through IL-7-STAT3-TCF1-Blimp1 axis. Expression and quantification of p-STAT3^Y705^ in pMKO.1/sh*Snhg1*/sh*Vps13D* GFP^+^ P14 CD8 T cells on day 15 p.i. **b**, **c** ChIP assay of Rabbit anti- p-STAT3^Y705^, p-STAT3^S727^and p-STAT5^Y694^ to the indicated genes, compared with normal Rabbit IgG, in CD8 T cells stimulated with IL-7. **d** Flow cytometry of the proportion of TCF-1^+^ cells in pMKO.1/sh*Snhg1*/sh*Vps13D* GFP^+^ P14 CD8 T cells on day 35 p.i. **e** Expression and quantification of TCF-1 in pMKO.1/sh*Snhg1*/sh*Vps13D* groups on day 35 p.i. **f** MFI of total CD127 (staining with cell punching buffer) in pMKO.1/sh*Snhg1*/sh*Vps13D* groups on day 15 p.i. **g** ChIP assay of Rabbit anti- TCF-1 to the indicated genes, compared with normal Rabbit IgG, in CD8 T cells stimulated with IL-7. **h**, **i** The established GFP^+^ P14 CD8 T cells were sorted as indicated (**h**) for RT-qPCR analysis of indicated genes on day 17 p.i. (**i**). **j**, **k** LCMV-infected mice with pMKO.1/sh*Snhg1*/sh*Vps13D* (IRES-*GFP*) and pMIT/*Tcf7*/*Bcl2* (IRES*-Thy1.1*) co-transducted together as indicated (**j**). FACS analysis of CD127^hi^KLRG1^lo^ cells in double-positive (GFP^+^Thy1.1^+^) P14 CD8 T cells were performed on day 35 p.i. (**k**). Data are representative of two or three independent experiments with at least three replicates or four mice per group (error bars denote s.e.m.). ns, not significant; *p < 0.05, **p < 0.01, ***p < 0.001, ****p < 0.0001 (unpaired two-tailed *t*-test). See also Fig. S6, Tables S1, 3

It was thought IL-7 mainly activates BCL-2 through STAT5 for memory T cell survival in memory CD8 generation.^3^ However, apoptosis of CD8 effector cells during the declining phase is not prevented by constitutive expression of BCL-2 in T cells; and the generation of T cell memory was not influenced.^30,31^ In short, IL-7 was found to sustain memory survival through STAT5-BCL-2 axis, but whether IL-7 can directly promote memory differentiation remain cloudy. There seems to exist certain transcription factors unrevealed downstream of IL-7 that can promote memory CD8 differentiation. We supposed IL-7 plays a role in memory differentiation through certain transcription factors, which maybe STAT3, because the cytokine-JAK-STAT signaling may also trigger STAT3, which was reported to play important role in cell differentiation.^32^ As the cytokine-JAK-STAT3 signaling mainly promotes STAT3 phosphorylation on Y705 primarily,^33^ we assessed the MFI of p-STAT3^Y705^ and found it can be impaired by Snhg1/Vps13D depletion in memory CD8 T cells stimulated with IL-7 (Fig. 6a). Since the transcription factors TCF-1, BCL-6 and Blimp-1 were found to play important roles in CD8 T cell differentiation and IL-7Rα deletion will result in severe memory deficiency,^1,2,6^ we first tried ChIP assay in CD8 T cells stimulated with IL-7, and found the *Tcf7* 5′-regulatory region can be regulated by direct binding of both p-STAT3^Y705^ (stronger) and p-STAT3^S727^ but not p-STAT5^Y694^. At the same time, we found *Il7r* gene also can be regulated by p-STAT3^Y705^ as a feedback loop of IL-7-STAT3 signaling, whereas not by p-STAT3^S727^ or p-STAT5^Y694^. Meanwhile, we found *Bcl6*, *Prdm1* genes were neither regulated by p-STAT3 nor p-STAT5 (Fig. 6b, c).

Moreover, both frequency and MFI for TCF-1 were impaired following loss of Snhg1/Vps13D, both externally and internally (Fig. 6d, e, Supplementary Fig. S6a). Meanwhile, we tested the total MFI of IL-7Rα using cell punching buffer for staining, and found IL-7Rα had a totally lower protein level as a feedback loop on day 15 post infection (Fig. 6f), although not as dramatic as that of membrane MFI change. Further, we found *Prdm1* rather than *Bcl6* can be regulated by TCF-1 with direct binding to its 5′-regulatory region in CD8 T cells stimulated with IL-7 (Fig. 6g). Although we found gene *Bcl6* neither can be directly regulated by p-STAT3 nor TCF-1, Blimp-1 (gene *Prdm1*) was reported to inhibit each other’s gene expression with BCL-6,^34^ and Blimp-1 also can repress gene *Id3* expression.^35^ Therefore, our data indicated the Snhg1/Vps13D regulated IL-7-STAT3-TCF-1 axis can promote memory with blocking effector mainly through direct impeding *Prdm1* expression to balance Blimp1-BCL6 and Blimp1-ID3 axis.

Furthermore, we examined the transcriptome which might be altered by p-STAT3-TCF-1. Using the established sh*Snhg1*/sh*Vps13D* (IRES-GFP) retrovirus transducted cells and LCMV-infected mice (Fig. 6h), the RT-qPCR analysis from the sorted GFP^+^ P14 cells in the contraction phase (on day 17 p.i.) showed that the *Prdm1, Granzyme B*, and *Klrg1* transcripts were enhanced while transcripts of *Tcf7, Bcl6, Id3, Eomes, Il7r, Cd27, Sell, Ccr7* and *Foxo1* were impaired, with no apparent changes of *Tbx21, Id2, Il2r* under conditions of Snhg1/Vps13D depletion (Fig. 6i). While there is no antibody distinguishing the two isoforms of TCF-1 at protein level, we designed primers that can specifically detect *p45* (the longer one). The results show in Fig. 6i that, while *Tcf7* can be regulated by Snhg1/Vps13D deficiency, *p45* is not affected, which may be caused by the different 5′ UTR. In the mean time, we confirmed the knockdown efficiency of Snhg1/Vps13D and found that Vps13D can somehow influence Snhg1 expression level (Fig. 6i), which explains the finding that Vps13D showed a greater effects than Snhg1. As a mutual confirmation, we detected the protein levels of several genes for proteome analysis. Consistently, with the increase of KLRG1 and GzmB, the protein levels of EOMES, CD27, CD62L, and IL-7Rα all decreased both externally and internally with loss of Snhg1/Vps13D (Supplementary Fig. S6c, h). We furthermore inspected the transcriptome on day 45 post-infection in the maintenance stage. At this stage, memory cells seem to enter a resting state, with no obvious changes in the transcripts associated with cell differentiation (Supplementary Fig. S6b). As a result, Snhg1/Vps13D depletion impairs the transcriptional launch program of memory CD8 T cells mainly in the expansion and contraction phases.

To verify that TCF-1 is directly downstream of and regulated by Snhg1/Vps13D, we established LCMV-infected mice with pMKO.1/sh*Snhg1*/sh*Vps13D* (IRES-GFP) and pMIT/*Tcf7*/*Bcl2* (IRES-Thy1.1) co-transducted (Fig. 6j). FACS analysis of double-positive (GFP^+^Thy1.1^+^) P14 CD8 T cells were performed on days 9, 18, and 35 post infection. The results revealed that TCF-1, especially the short isoform p33 (two isoforms of TCF-1, p33 and p45), rescued the Snhg1/Vps13D depletion phenotype both in the expansion and contraction phases. As a contrast, overexpressed *Bcl2* cannot save this process (Fig. 6k, Supplementary Fig. S6d). In other words, IL-7 promotes memory generation not just because of the sustaining role of BCL-2. For further verification, we also tested and found impairment of CD27 by Snhg1/Vps13D deprivation can also be rescued by TCF-1, whereas not by BCL-2 (Supplementary Fig. S6e). At the same time, we double-checked and confirmed the expression of BCL-2 and TCF-1, both of them with an expression no problem (Supplementary Fig. S6f). Interestingly, we found transgenic TCF-1 can induce Snhg1 especially Vps13D expression as a feedback loop (Supplementary Fig. S6g).

### The IL-7 signaling regulated by Snhg1-Vps13D in memory CD8 generation shows the dual-function and dose-effect

**Fig. 7:**
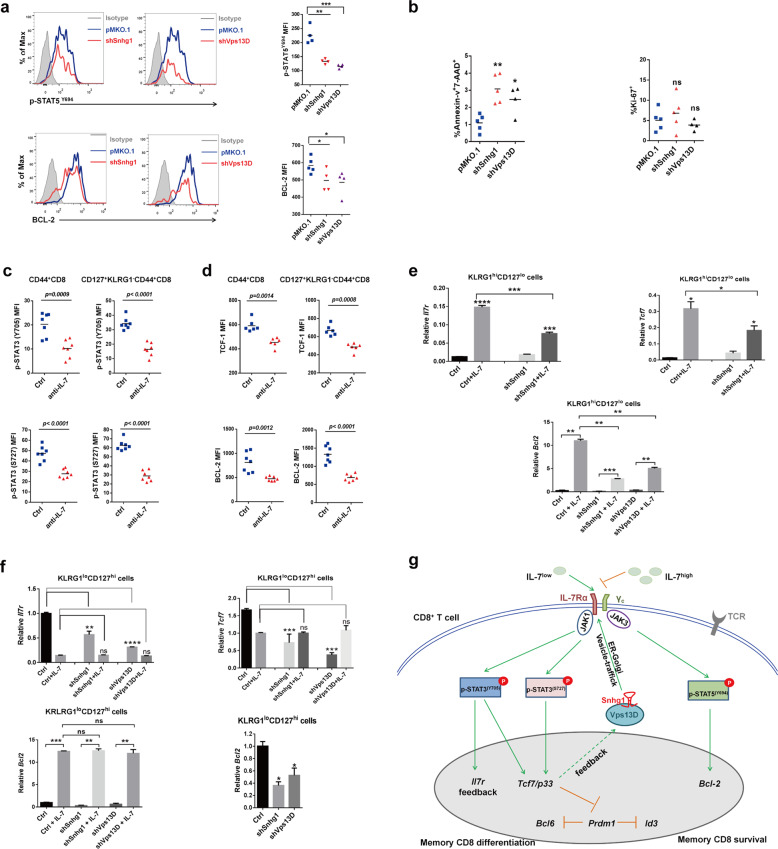
The dual-function and dose-effect of Snhg1-Vps13D regulated IL-7 signaling in memory CD8 T cell establishment. **a** Mean fluorescence intensity of p-STAT5^Y694^ and BCL-2 in control or sh*Snhg1*/sh*Vps13D* GFP^+^ P14 cells on day 45 p.i. **b** The proportion of Annexin-v^+^7-AAD^+^ and Ki-67^+^ cells in GFP^+^ P14 cells on day 40 p.i. **c**, **d** C57 mice were injected (i.v.) with PBS or 100 ug anti-IL-7 mAb at 2 days intervals since day 8 p.i., MFI of p-STAT3 (Y705/S727)/TCF-1/BCL-2 in bulk memory (left) for memory T cells (right) were analyzed on day 15 p.i. **e**–**f** The sorted CD127^hi^ or CD127^lo^ cells from pMKO.1/sh*Snhg1*/sh*Vps13D* groups on day 8 p.i. were treated with PBS or 5 ng/ml IL-7 for 48 h culturing in vitro followed by RT-qPCR analysis of *Il7r/Tcf7/Bcl2*. **g** A model to show the dual-function and dose-effect of Snhg1-Vps13D regulated IL-7 signaling in memory CD8 T cell establishment

Finally, to elucidate the role of Snhg1 and Vps13D in memory CD8 T cell maintenance, we inspected MFI (mean fluorescence intensity) of BCL-2, Annexin-V, Ki-67, and p-STAT5^Y694^ (stimulated with IL-7 to catch the phosphorylation state of STAT5) in memory CD8 T cells, for memory survival and self-renewal analysis on day 45 post-infection. The results showed that Snhg1 and Vps13D depletion impaired memory survival with increasing effector T cell apoptosis (Fig. 7a, b) to a certain degree in the maintenance stage (day 40), without affecting cell proliferation (Fig. 7b). Furthermore, to verify that p-STAT3-TCF-1 can genuinely affected memory formation through IL-7 signaling, we firstly applied IL-7 in vivo ablation experiments from day 8 post-infection, and found both Y705 and S727 of p-STAT3 were reduced obviously in both bulk memory (CD44^+^ CD8) and CD127^+^KLRG1^−^ memory CD8 T cells on day 15 post infection (Fig. 7c). Moreover, both TCF-1 and BCL-2 were impaired in memory CD8 T cells in the condition of IL-7 ablation (Fig. 7d). We also applied IL-7 in vitro induction experiments, which showed that IL-7 can greatly induce *Il7r* with *Tcf7* and *Bcl2* in the sorted CD127^lo^ cells on day 8, and Snhg1/Vps13D depletion can impair this induction (Fig. 7e). Meanwhile, Snhg1/Vps13D depletion can also impair *Il7r* with *Tcf7* and *Bcl2* in the sorted CD127^hi^ cells. But when treated with additional IL-7 (5 ng/ml), the CD127^hi^ cells seem to generate a feedback to the high IL-7-CD127 signal with controlling *Il7r, Tcf7*, and *Bcl2* at a constant level (Fig. 7f). As a result, IL-7 shows to be such important to exist a dose-effect, which may shut down IL-7R with TCF-1, when it’s too high to block an excess of memory formation. In summary, the above results indicated that the IL-7 signaling regulated by Snhg1-Vps13D in memory CD8 generation shows the dual-function and dose-effect, including not just cell survival, but also cell differentiation (Fig. 7g).

## Discussion

The efficient induction and long-term persistence of pathogen-specific memory CD8 T cells are pivotal to rapidly curb the reinfection. Recent evidence indicated that lncRNAs expression is highly cell-lineage and cell-stage specific during T cell development and differentiation, suggesting their potential roles in regulating T cell programs. Here through CD8 T cell subsets profiling of lncRNAs, this study shows a key lncRNA-Snhg1 with the conserved naive^hi^-effector^lo^-memory^hi^ expression pattern from mouse to human, that can promote memory, especially central memory CD8, differentiation while impeding effector CD8 T cells in acute viral infection. Further, Snhg1 was found interacting with the conserved vesicle trafficking protein Vps13D to facilitate IL-7Rα membrane location specifically. With the deep mechanism probing, the results show Snhg1-Vps13D regulated IL-7 signaling with the dual-function in memory CD8 generation, which not just because of the sustaining role of STAT5-BCL-2 axis for memory survival, but more through the STAT3-TCF1-Blimp1 axis for the transcriptional launch program of memory differentiation. Figure 7g is the working model to show the dual-function and dose-effect of Snhg1-Vps13D regulated IL-7 signaling in memory CD8 T cell establishment. On the other hand, given the competitively and synergetic regulatory role between STAT3 and STAT5 downstream of IL-7, our data suggested that both of them are necessary for the memory transition stage, while the STAT5-BCL-2 survival axis plays an additional role in the memory maintenance stage. For the interaction and regulation between Snhg1, Vps13D, and IL-7R, our results suggested that the lncRNA Snhg1 might play a guiding role for recruiting its target IL-7R to the Vps13D vesicle trafficking system, which can then promote IL-7R membrane location, as evidenced by that both Vps13D and IL-7Rα can pull down Snhg1 in RIP assay, and IL-7Rα had colocalization with Vps13D, which can be obviously reduced by Snhg1 depletion in memory CD8 T cells. In contrast, CD122/IL-2Rβ did not show any colocalization with Vps13D regardless of Snhg1 presence or not. What’s more, Snhg1/Vps13D also does not affect the common gamma chain CD132 membrane location. In fact, the ncRNAs which occupied more than 80% of human genome, in most cases play a more specific role than the housekeeping mRNAs (~2% of genome).^11,12^ On the other hand, Vps13D has the repeating coiled-coil domain with the N-terminal Chorein-N domain containing leucine zipper, which may form dimer and be reasonable for the binding of VPS13D with RNAs.^36^ Vps13D also has a UBA domain that contains two putative domains, ubiquitin-associated (UBA) domain and lectin domain of ricin B chain profile (ricin-B-lectin), suggesting it may interact with, and be involved in the trafficking of, proteins modified with ubiquitin and/or carbohydrate molecules.^18,19^

It was thought IL-7 mainly activates BCL-2 through STAT5 for cell survival during memory generation in the previous study.^3^ Whether IL-7 can directly promote memory differentiation through transcription factors remain unclear. In this study, we found the *Tcf7* 5′-regulatory region can be regulated by direct binding of both p-STAT3^Y705^ (stronger) and p-STAT3^S727^ but not p-STAT5^Y694^ in CD8 T cells stimulated with IL-7 for memory differentiation. Since STAT3 acts as a central transcription factor downstream of cytokines and thus regulates antimicrobial responses and cell differentiation, impaired STAT3 function results in immunodeficiency and in some cases tumorigenesis. The dominant-negative STAT3 mutations result in the Hyper-IgE syndrome (HIES) characterized by elevated IgE levels, eczema, recurrent staphylococcal skin and pulmonary infections and pleiotropic somatic manifestations, including the systemic lupus erythematosus (SLE) or SLE-like manifestations which are characterized by aberrant type I interferon (IFN) responses with increased expression of IFN-stimulated genes (ISGs) in blood and affected tissues.^37,38^ Although STAT3 can be regulated by IL-21, IL-21 knockout mice did not show any phenotype on memory CD8 formation.^39^ Moreover, IL-21 was found playing more important roles in exhausted CD8 T cell function.^40,41^ For this study, we applied IL-7 in vivo ablation experiments and found both p-STAT3 and TCF-1 were reduced obviously in memory CD8 T cells. We also applied IL-7 in vitro induction experiments, which showed that IL-7 can induce *Il7r* and its downstream *Tcf7*/*Bcl2*, with all of them showing a dose-effect. Moreover, Snhg1/Vps13D depletion can impair this induction. Although there was a report about that IL-7 inhibited TCF-1 expression in thymocyte development,^42^ they used 60 ng/ml IL-7 treated fetal thymic lobes for 6 days in vitro, which is really too high levels of IL-7 to mimic the real condition of IL-7 in vivo. Meanwhile, there was a study showed the dose-effect of IL-7 for thymocyte development in vivo. They generated IL-7 gene transgenic mice (Tg) under the control of *lck* promoter. The founder line-TgA with the lowest level of IL-7 overexpression, showed enhanced T-cell development. In contrast, the founder line-TgB with the highest overexpression, showed disturbed T-cell development with a block at the earliest intrathymic precursor stage.^43^ Collectively, our study suggested that IL-7 is such important to show a dose-effect in memory CD8 generation, which may shut down cell differentiation with controlling IL-7R/TCF-1 levels, when it’s too high to block an excess of memory formation.

The transcription factor TCF-1 was found to play a key role in memory CD8 T cell differentiation from mouse to human.^4–6^ However, whether TCF-1 is induced by canonical Wnt-β-Catenin signaling in memory CD8 generation remains controversial. And the mechanism of how TCF-1 promotes memory with blocking effector remains to be elucidated. Here, we verified that TCF-1 is directly downstream of and regulated by IL-7/Snhg1/Vps13D with promoting memory and impeding effector CD8 mainly through balancing Blimp1-BCL6 and Blimp1-ID3 axis. Moreover, we found the short isoform p33 was much more effective than the long isoform p45. And our results show that, while *Tcf7* can be regulated by Snhg1/Vps13D deficiency, *p45* is not affected, which may be caused by the different 5′ UTR. For why p45 still work to some extent, the overexpressed p45 might play some role as the way of p33, which is independent of β-Catenin in memory CD8 T cells. Furthermore, we found *Prdm1* rather than *Bcl6* can be regulated by TCF-1 in CD8 T cells stimulated with IL-7. Although *Eomes* and *CD122* (*Il2rb*) can be bound by TCF-1, they were verified to mainly play a sustaining role for later memory persistence.^6^ Blimp-1 (gene *Prdm1*) was reported to inhibit each other’s expression with BCL-6,^44^ and Blimp-1 can also repress *Id3* expression.^35^ Therefore, the above data suggested that the Snhg1-Vps13D regulated IL-7-STAT3-TCF-1 axis can block effector with promoting memory program mainly through direct impeding *Prdm1* expression by the isoform p33, to balance Blimp1-BCL6 and Blimp1-ID3 axis. Actually, it’s not surprising for the short isoform TCF1–p33 to promote memory with impeding effector T cells, as its dominate negative role with only the transcription-repressor binding domain while with lacking the transcription-activator binding domain,^45^ to inhibit *Prdm1* expression, since Blimp-1(gene *Prdm1*) plays a major role in promoting effector while inhibiting memory formation. Finally, the above study suggested that TCF-1 can function as a repressor of effector CD8 T cells with promoting memory CD8 T cell formation independent of the canonical Wnt-β-Catenin signaling, which play key roles in early development and early T cell maturation, and whose ectopic activation would cause cancer in adults.^46^

LncRNAs are known to show more cell-type specificity than protein-coding mRNAs. Dysregulated expression of lncRNAs has been demonstrated as being implicated in a variety of human diseases. In autoimmune and immune-related disorders (AID), more than 90% of the risk variants lie in non-coding regions, and almost 10% of these map to lncRNAs;^47^ additionally, 328 lncRNAs were aberrantly expressed in tuberculosis (TB) CD8 T cells;^48^ Moreover, CD8 T cells expanding in response to primary infection by LCMV differentially expressed more than 800 lncRNAs, of which more than 500 were unannotated novel genes.^49^ The topic of research of lncRNAs in CD8 T cells is still very young and the function of most of them is not known to date. Further studies should focus on the function of lncRNAs involved to interpret GWAS (genome-wide association studies) findings. We also compared our profiling dataset with that in Hudson et al’s report, finding the great correlation between the two studies with regard to both lncRNAs and mRNAs expression patterns (Supplementary Fig. S6j), and particularly, Snhg1 also shows the naive^hi^-effector^lo^-memory^hi^ expression pattern in Hudson et al’s profiling (Supplementary Fig. S6k). In short, this study provides novel insights in the role of lncRNAs in T cell differentiation upon virus infection, establishing a model for lncRNA Snhg1 as a critical regulator orchestrating the cytokine induction with transcriptional launch program in memory CD8 T cell generation.

What’s more, as the global Coronavirus disease 2019 (COVID-19) pandemic caused by SARS-CoV-2 that is rapidly spreading across corners has changed the world, we performed further study giving an insight into the expression level of human *SNHG1/VPS13D/IL7R/TCF7* in CD8 T cell subsets from PBMC samples of the convalescent COVID-19 patients in China, with finding their similar high-low-high expression pattern in the human naive-Teff-Tcm/Tem/Temra cells (Fig. S6i). As the acute phase of COVID-19 in humans is associated with strong T cell lymphopenia in severe disease with a bias towards CD8 T cells,^50,51^ the central role of Snhg1-Vps13D-IL-7R-TCF1 axis in memory CD8 establishment makes it a potential target for improving the vaccination effects to control the ongoing pandemic.

## Methods with materials

For full “Methods with materials”, please refer to the Supplementary Methods with Materials.

## Supplementary information

Supple Inform

## Data Availability

The source for the lncRNA-seq & mRNA-seq data of naive-effector-memory CD8 T cell profiling (upon acute LCMV infection) reported in this paper is SRA accession: PRJNA592025; and the source for the mRNA-seq data of sh*Snhg1*/sh*Vps13D*/pMKO.1 transducted memory CD8 T cells (sorted from adoptive transferred mice) is SRA accession: PRJNA592213.

## References

[1] Kaech SM, Cui W (2012). Transcriptional control of effector and memory CD8 + T cell differentiation. Nat. Rev. Immunol..

[2] Chang JT, Wherry EJ, Goldrath AW (2014). Molecular regulation of effector and memory T cell differentiation. Nat. Immunol..

[3] Kurtulus S (2011). Bcl-2 allows effector and memory CD8+ T cells to tolerate higher expression of Bim. J. Immunol..

[4] Weber BN (2011). A critical role for TCF-1 in T-lineage specification and differentiation. Nature.

[5] Gullicksrud J (2017). Differential requirements for Tcf1 long isoforms in CD8^+^ and CD4^+^ T cell responses to acute viral infection. J. Immunol..

[6] Zhou X (2010). Differentiation and persistence of memory CD8+ T cells depend on T cell factor 1. Immunity.

[7] Prlic M, Bevan MJ (2011). Cutting edge: β-catenin is dispensable for T cell effector differentiation, memory formation, and recall responses. J. Immunol..

[8] Tiemessen MM (2014). T cell factor 1 represses CD8+ effector T cell formation and function. J. Immunol..

[9] Atianand MK, Caffrey DR, Fitzgerald KA (2017). Immunobiology of long noncoding RNAs. Annu. Rev. Immunol..

[10] Heward JA, Lindsay MA (2014). Long non-coding RNAs in the regulation of the immune response. Trends Immunol..

[11] Hu GQ (2013). Expression and regulation of intergenic long noncoding RNAs during T cell development and differentiation. Nat. Immunol..

[12] Jia H (2010). Genome-wide computational identification and manual annotation of human long noncoding RNA genes. RNA.

[13] B B (2007). Analysis of CD127 and KLRG1 expression on hepatitis C virus-specific CD8+ T cells reveals the existence of different memory T-cell subsets in the peripheral blood and liver. J. Virol..

[14] Youngblood B (2017). Effector CD8 T cells dedifferentiate into long-lived memory cells. Nature.

[15] Akondy RS (2017). Origin and differentiation of human memory CD8 T cells after vaccination. Nature.

[16] D’Asaro M (2006). Increase of CCR7− CD45RA+ CD8 T cells (TEMRA) in chronic graft-versus-host disease. Leukemia.

[17] Chaudhry MA (2013). Expression pattern of small nucleolar RNA host genes and long non-coding RNA in X-rays-treated lymphoblastoid cells. Int. J. Mol. Sci..

[18] Anding AL (2018). Vps13D encodes a ubiquitin-binding protein that is required for the regulation of mitochondrial size and clearance. Curr. Biol..

[19] Velayosbaeza A (2004). Analysis of the human VPS13 gene family. Genomics.

[20] Wang Z, Zhang H (2018). Mitophagy: Vps13D couples mitochondrial fission and autophagic clearance. Curr. Biol..

[21] Bean BDM (2018). Competitive organelle-specific adaptors recruit Vps13 to membrane contact sites. J. Cell Biol..

[22] Mccausland MM, Crotty S (2008). Quantitative PCR technique for detecting lymphocytic choriomeningitis virus in vivo. J. Virolog. Methods.

[23] Alpdogan O, Den Brink MRMV (2005). IL-7 and IL-15: therapeutic cytokines for immunodeficiency. Trends Immunol..

[24] Cieri N (2013). IL-7 and IL-15 instruct the generation of human memory stem T cells from naive precursors. Blood.

[25] Nanjappa SG, Walent JH, Morre M, Suresh M (2008). Effects of IL-7 on memory CD8+ T cell homeostasis are influenced by the timing of therapy in mice. J. Clin. Investig..

[26] Schluns KS, Kieper WC, Jameson SC, Lefrançois L (2000). Interleukin-7 mediates the homeostasis of naïve and memory CD8 T cells in vivo. Nat. Immunol..

[27] Kieper WC (2002). Overexpression of interleukin (IL)-7 leads to IL-15–independent generation of memory phenotype CD8+ T cells. J. Exp. Med..

[28] Rubinstein MP (2008). IL-7 and IL-15 differentially regulate CD8+ T-cell subsets during contraction of the immune response. Blood.

[29] Schluns KS, Lefrançois L (2003). Cytokine control of memory T-cell development and survival. Nat. Rev. Immunol..

[30] Petschner F (1998). Constitutive expression of Bcl‐xL or Bcl‐2 prevents peptide antigen‐induced T cell deletion but does not influence T cell homeostasis after a viral infection. Eur. J. Immunol..

[31] Hand TW, Morre M, Kaech SM (2007). Expression of IL-7 receptor α is necessary but not sufficient for the formation of memory CD8 T cells during viral infection. Proc. Natl Acad. Sci. USA.

[32] Levy DE, Lee C (2002). What does Stat3 do. J. Clin. Investig..

[33] Schuringa JJ, Wierenga ATJ, Kruijer W, Vellenga E (2000). Constitutive Stat3, Tyr705, and Ser727 phosphorylation in acute myeloid leukemia cells caused by the autocrine secretion of interleukin-6. Blood.

[34] Johnston RJ (2009). Bcl6 and Blimp-1 are reciprocal and antagonistic regulators of T follicular helper cell differentiation. Science.

[35] Ji Y (2011). Repression of the DNA-binding inhibitor Id3 by Blimp-1 limits the formation of memory CD8+ T cells. Nat. Immunol..

[36] Nikolaev Y, Pervushin K (2012). Structural basis of RNA binding by leucine zipper GCN4. Protein Sci..

[37] Holland SM (2007). STAT3 mutations in the hyper-IgE syndrome. N. Engl. J. Med..

[38] Goel RR (2020). Lupus-like autoimmunity and increased interferon response in patients with STAT3-deficient hyper-IgE syndrome. J. Allergy Clin. Immunol..

[39] Cui W (2011). An interleukin-21-interleukin-10-STAT3 pathway is critical for functional maturation of memory CD8+ T cells. Immunity.

[40] Sutherland APR (2013). IL-21 promotes CD8+ CTL activity via the transcription factor T-bet. J. Immunol..

[41] Tian Y, Zajac AJ (2016). IL-21 and T cell differentiation: consider the context. Trends Immunol..

[42] Yu Q (2004). IL-7 receptor signals inhibit expression of transcription factors TCF-1, LEF-1, and RORgammat: impact on thymocyte development. J. Exp. Med..

[43] Kassar NE (2004). A dose effect of IL-7 on thymocyte development. Blood.

[44] Xu L (2015). The transcription factor TCF-1 initiates the differentiation of T FH cells during acute viral infection. Nat. Immunol..

[45] Zhu Y, Wang W, Wang X (2015). Roles of transcriptional factor 7 in production of inflammatory factors for lung diseases. J. Transl. Med..

[46] Gattinoni L, Ji Y, Restifo NP (2010). Wnt/β-catenin signaling in T-cell immunity and cancer immunotherapy. Clin. Cancer Res..

[47] Hrdlickova B (2014). Expression profiles of long non-coding RNAs located in autoimmune disease-associated regions reveal immune cell-type specificity. Genome Med..

[48] Fu Y (2017). Aberrantly expressed long non-coding RNAs in CD8+ T cells response to active tuberculosis. J. Cell. Biochem..

[49] Hudson WH (2019). Expression of novel long noncoding RNAs defines virus-specific effector and memory CD8+ T cells. Nat. Commun..

[50] Chen Z, John Wherry E (2020). T cell responses in patients with COVID-19. Nat. Rev. Immunol..

[51] Gao, L. et al. The dichotomous and incomplete adaptive immunity in COVID-19. Reprint at https://www.medrxiv.org/content/10.1101/2020.09.05.20187435v1 (2020). 10.1038/s41392-021-00525-3 PMC7938043 33686064

