# Supplementary material for: The lncRNA Snhg1-Vps13D vesicle trafficking system promotes memory CD8 T cell establishment via regulating the dual effects of IL-7 signaling
Source: Signal Transduct Target Ther. 2021 Mar 24;6:126. doi: 10.1038/s41392-021-00492-9 (PMC7987995; doi:10.1038/s41392-021-00492-9)
Supplement: Supplementary file 1 — Supple Inform [file 41392_2021_492_MOESM1_ESM.pdf]

Supplementary Materials for

The lncRNA Snhg1-Vps13D vesicle trafficking system promotes memory CD8 T cell establishment via regulating the dual effects of IL-7 signaling

Yanyan Zhang*, Baohua Li, Qiang Bai, Pengcheng Wang, Gang Wei, Zhirong Li, Li Hu, Qin Tian, Jing Zhou, Qizhao Huang, Zhiming Wang, Shuai Yue, Jialin Wu, Liuqing Yang, Xinyuan Zhou, Ting Ni, Lubin Jiang, Lilin Ye* & Yuzhang Wu*

*Correspondence to: yzhangtmmu@163.com (Y.Z.); yelilinlcmv@tmmu.edu.cn (L.Y.); wuyuzhang@tmmu.edu.cn (Y.W.)

This PDF file includes:

Methods with Materials.

Supplementary Figures. S1 to S6.

Supplementary Tables. S1 to S6.

Methods with Materials

Mice and virus infection

The P14 TCR transgenic mice (CD45.1) were obtained from Dr. Rafi Ahmed (Emory university). C57BL/6J (CD45.2) mice were from the Jackson Laboratories. All mice analyzed were 6-10 weeks of age, and both genders were included without randomization or blinding. The Lymphocytic choriomeningitis virus (LCMV) Armstrong was provided by Dr. Rafi Ahmed, and 2×10^5 plaque-forming units (PFU) were used to establish acute infection in mice with i.p.. Mice infected with LCMV were housed in accordance with Institutional Biosafety Regulations of Third Millitary Medical University. All mouse experiments were performed in accordance with the guidelines of Institutional Animal Care and Use Committees of Third Millitary Medical University.

Adoptive transfer and cell sorting

In each individual experiment that if did not need to quantify cell number after infection, a total of ~2-5$\times 10^4$ naïve $CD45.1^+$ P14 cells with retrovirus transduction (~2-5,000 GFP^+ cells dependent on transduction efficiency) were adoptively transferred into naïve wild-type ($CD45.2^+$) mice, and infected recipient mice with 2×10^5 PFU LCMV Armstrong i.p. on the following day. For cell number quantification experiments, the cell sorting was performed on a FACSAria Ⅲ (BD Biosciences) with 2000 GFP^+ cells injected for each mouse, and the purity of all sorted populations was > 95%.

Flow cytometry and antibodies

Flow cytometry data were acquired by FACSCantoII (BD Biosciences) and analyzed with FlowJo software (Tree Star). The antibodies and reagents used for flow cytometry staining are listed in Supplementary Table S1. Surface staining was performed in PBS containing 2% BSA or FBS (wt/vol). Intracellular staining of TCF-1, Eomes and Ki67 were performed with the Foxp3/Transcription Factor Staining Buffer Set (00-5523; eBioscience). Staining of BCL-2 was performed with a Cytofix/Cytoperm Fixation/Permeabilization Kit (554722; BD Biosciences). Annexin V staining were performed with an Annexin V Kit (88-8102-72; eBioscience) according to the manufacturer's instructions. For detection of cytokine production, splenocytes were first stimulated with the indicated peptide (0.2 μg/ml), Golgi Plug, Golgi Stop, anti-CD107a, and anti-CD107b antibodies (BD Biosciences) at 37°C for 5 hrs. Following surface staining, intracellular cytokine staining was performed with a Cytofix/Cytoperm Fixation/Permeabilization Kit according to the manufacturer's instruction. For detection of phosphorylated STAT signaling proteins, splenocytes were first stained with surface markers and then were stimulated with recombinant murine IL-7 (2 ng/ml, 217-17; PeproTech) at 37°C for 1 hr. Then immediately fixed the cells with Phosflow Lyse/ Fix buffer (558049; BD Biosciences), followed by permeabilization with Phosflow Perm buffer I (557885; BD Biosciences) and staining with primary unconjugated antibodies to STAT3 (Tyr705) or STAT5 (Tyr694). Afterwards, primary unconjugated antibodies were detected by secondary staining with anti-rabbit IgG A647 antibody.

Retroviral constructs and transduction

The constructs pMKO.1 (IRES-*GFP*) and pMIT (IRES-*Thy1.1*) were obtained from Dr. Rafi Ahmed. The shRNA sequences targeting Snhg1 or Vps13D were cloned into vector pMKO.1, and the shRNA used here were listed in Table S3. The *Bcl2* and *Tcf7* coding sequences (p33 and p45 isoform) were amplified and cloned into the vector pMIT. Retroviruses were packaged by transfection of 293T cells with the retroviral vectors along with plasmid pCLeco. P14 CD8 T cells were activated in vivo by injection of 200μg peptide (LCMV glycoprotein amino acids 33-41) into P14 mice. 16-18 hrs later, activated P14 CD8 T cells were isolated and purified by negative selection with BeaverBeads Mag500 Streptavidin Matrix (22302, Beaver) , and then spin-infected for 120 min at 37°C with centrifugation (800g) in freshly harvested retrovirus supernatants containing 8 μg/ml polybrene (H9268, Sigma-Aldrich) and 20 ng/ml of IL-2 (130-098-221, Miltenyi Biotec). Then, the transducted P14 cells were transferred into recipient mice, followed by infection of the hosts with LCMV Arm 12-16 hrs later.

Quantitative RT-PCR

For comparison of gene expression in P14 cells transducted with retrovirus expressing empty vector or Snhg1/Vps13D shRNA, the cells were sorted by a FACSAria Ⅲ cell sorter (BD Biosciences) and total RNA was extracted using the RNeasy Plus Mini Kit (74134; QIAGEN), thus reverse transcribed with a RevertAid H Minus First Strand cDNA Synthesis Kit (K1632; Thermo Scientific). The relative expression of various genes was examined using AceQ qPCR SYBR Green Master Mix (Q111; Vazyme) on a CFX96 Touch Real-Time System (Bio-Rad). The primers for the test genes are listed in Table S3.

Immunofluorescence staining

Briefly, the enriched CD8 T cells from the adoptive transferred mice were fixed and permeablized, and then stained using Rabbit anti-Vps13D (ab202285, Abcam) with Rat anti-CD122 (ab61195, Abcam) or Goat anti-CD127 (ab180521, Abcam) on day 35 p.i. in the presence of shSnhg1 (GFP) or not; Rabbit anti-coatomer (ab96725, Abcam) or Rabbit anti-Golgin160 (HPA040044, Merck) with Goat anti-CD127 in the presence of shSnhg1/shVps13D (GFP) or not on day 20 p.i.. The secondary antibody used A647 Goat anti-Rabbit or anti-Rat IgG (green), A555 Donkey anti-Goat IgG or Goat anti-Rabbit IgG (red). Finally, DAPI (4,6-diamidino-2-phenylindole; D9542; Sigma-Aldrich) was added to define the nucleus. Coverslips were mounted on slides using the ProLong Antifade Kit (P-7481; Life Technologies), and then cells were examined by Zeiss LSM 510 confocal fluorescence microscope. The images were quantified with Image J software.

RNA-seq and bioinformatic analysis

For cell sorting of GFP^{+} P14 cells, total splenocytes from recipient mice (CD45.2) previously adoptively transferred with P14 cells (CD45.1) transducted with retrovirus expressing empty vector (pMKO.1) or Snhg1/Vps13D shRNA on day 17 post LCMV infection, were subjected to lineage depletion by using biotin-conjugated antibodies (anti-CD4 (RM4-5), anti-B220 (RA3-6B2), anti-CD11b (M1/70), anti-CD11c (N418), anti-TER119 (TER-119) and anti-NK1.1 (PK136); all from BioLegend), followed by coupling with BeaverBeads Mag500 Streptavidin Matrix (22302; Beaver). The enriched lineage marker-negative cells were then

stained with anti-CD8, anti-CD45.1 and anti-CD44 (all identified in Supplementary Table S1). For isolation of naïve, effector and memory CD8 T cells (bulk), splenocytes from naïve mice, or from mice on days 8 or 30 p.i. with LCMV were processed as above described, and stained with anti-CD8, anti-CD44, anti-CD62L, anti-CD127 and anti-KLRG1 (all identified in Table S1). The $CD8^+CD44^+CD45.1^+GFP^+$ P14 cells, $CD8^+CD44^-CD62L^+$ naïve CD8 T cells, $CD8^+CD44^+KLRG1^+CD62L^-$ effector CD8 T cells and $CD8^+CD44^+CD127^+$ memory CD8 T cells were soretd by a FACSAria Ⅲ cell sorter (BD Biosciences) and then immediately lysed with TRIzol LS reagent (10296; Life Technologies), and total RNA was extracted according to the TRIzol reagent protocol and submitted to CapitalBio Corporation for RNA sequencing.

RNA immunoprecipitation (RIP)

RNA immunoprecipitation was performed using the Magna RIP™ RNA-Binding Protein Immunoprecipitation kit (Millipore). Briefly, the harvested El4 or Jurkat cells were washed with ice-cold PBS and resuspended on ice with RIP lysis buffer. A/G magnetic beads were incubated with normal Rabbit IgG (Millipore), Rabbit anti-Vps13D (ab202285, Abcam) or Rabbit anti-CD127 (Abcam) for 30 min at room temperature. Then the cell lysate was incubated with the antibody-coated magnetic beads for 4 hours or overnight at 4°C, and the immunoprecipitates were treated with Proteinase K Buffer (RIP Wash Buffer, 10 % SDS and proteinase K) at 55°C for 30 minutes to digest the protein. RNA was then extracted and quantitative RT-PCR was performed for further analysis.

RNA pulldown coupled with mass spectrometry

Full-length Snhg1 was cloned into the pGEX-3Z vector from Dr. Liuqing Yang (M.D. Anderson Cancer Centre), and then Snhg1 RNA (sense with antisense control) was transcribed in-vitro using the Biotin RNA labeling mix (Roche, 24552321) and T7 or SP6 RNA polymerase (Ambion) and purified by RNA Clean & Concentrator-5 (Zymo Research). The sorted memory CD8 T cells (~5 million, bulk) were freshly prepared using ProteaPrep Zwitterionic Cell Lysis Kit, Mass Spec Grade (Protea) with Anti-RNase, Protease/Phosphatase Inhibitor Cocktail, Panobinostat, and Methylstat, supplemented in the lysis buffer. The BcMag Monomer Avidin Magnetic Beads (Bioclone) were first prepared in accordance with manufacturer's instructions and then immediately subjected to RNA (20 ug) capture in RNA capture buffer for 30 min at room temperature with agitation. The RNAcaptured beads were washed once with NT2 buffer and incubated with 30 mg cell lysates diluted in NT2 buffer supplemented with 50 U/ml RNase inhibitor, 2 mM dithiothreitol, 30 mM EDTA, and 0.02 mg/ml Heparin for 4 hr at 4oC with rotation. The RNA-binding protein complexes were washed sequentially with NT2 buffer, NT2 high-salt buffer, NT2-KSCN buffer and PBS, thus eluted by 2 mM D-biotin in PBS. The eluted protein complexes were denatured, reduced, alkylated, and digested with immobilized trypsin (Promega), and samples were sent to Shanghai Applied Protein Technology Co. Ltd. for mass spectrometry analysis.

Chromatin immunoprecipitation (ChIP)

The enriched CD8 T cells were stimulated with IL-7 (10ng/ml) at 37°C for 3hrs. Then the treated cells were conducted with ChIP assay using the SimpleChIP Enzymatic Chromatin IP Kit (Magnetic Beads) (9003; Cell Signaling Technology), according to the manufacturer's

instructions. Chromatin fragments were immunoprecipitated by Rabbit anti-p-STAT3^{Y705} (9145; Cell Signaling Technology), anti-p-STAT3^{S727} (9134; Cell Signaling Technology), anti-p-STAT5^{Y694} (9314; Cell Signaling Technology) or the normal rabbit IgG (3900; Cell Signaling Technology) coupled with ChIP Grade Protein G Magnetic Beads (9006; Cell Signaling Technology). After purification of DNA with a PCR purification kit (28104; Qiagen), quantitative PCR was performed with primers (Supplementary Table S3) flanking the putative p-STAT3 or p-STAT5 binding sites.

Immunoprecipitation (IP)-based mass spectrometry

Immunoprecipitation was performed using the Sure BeadsTM Protein G magnetic beads (BioRad, 161-4023). Briefly, the harvested EL4 cells were lysed thoroughly in RIPA buffer (proteinase inhibitor added) with pipeting using BD ultra-fine needle on ice, thus with supersonics on ice for getting membrane proteins. After centrifugation, the lysate was incubated with rabbit anti-Vps13D (ab202285, Abcam) or normal rabbit IgG (Abcam) for 2 hrs at 4°C, followed by incubation with the BSA pre-blocked Protein G magnetic beads for 1 hr at 4°C. Wash the immunoprecipitated samples with RIPA buffer and RIPA high buffer, thus samples were eluted using 200ul/IP SDT buffer at 95°C for 10min, thus with beads removed and sent to Shanghai Applied Protein Technology Co. Ltd. for mass spectrometry analysis.

Nucleus-cytoplasmic fractionation

Nucleus-cytoplasmic fractionation experiment was conducted using the Cytoplasmic and Nuclear RNA Extraction Kit (NORGEN, 21000), according to the manufacturer's protocol.

Human PBMC isolation and *in-vitro* culture

Human peripheral blood mononuclear cells (PBMCs) were isolated with Ficoll (SigmaAldrich) gradient separation from peripheral blood of 4 healthy adult donors (Fig. 1a, 2a) or 3 convalescent COVID-19 patients (Supplementary Fig. S6i). The blood samples of COVID-19 patients were obtained from Chongqing Public Health Medical Center. The study received IRB approvals at Chongqing Public Health Medical Center (2020-023-01-KY). For the acquisition of human effector CD8 T cells, the sorted na ïve CD8 T cells (CCR7^{+}CD45RA^{-}) were stimulated with Dynabeads™ Human T-Activator CD3/CD28 (11161D, Thermo Fisher Scientific) for three days in RPMI 1640 with 10% FCS. The study was reviewed and approved by the Ethics Committee of Chongqing Public Health Clinical Center, Third Millitary Medical University. Written informed consents were offered to all study participants.

Cytokines and neutralizing Ab treatments

To examine the cytokines' roles on the regulation of gene expression, cells were incubated at 37°C with indicated concentrate of recombinant murine IL-7 (rmIL-7, PeproTech) or recombinant murine IL-2 (rmIL-2, PeproTech) for 48 hours in vitro, followed by RT-qPCR analysis. For depletion of IL-7, mice were treated on indicated days with 100 μg anti-IL-7 mAbs (M25, Bio X Cell) per day with i.v. injection in 500 μL PBS.

Virus titration

The LCMV viral loads in the spleen were quantified by RT-qPCR analysis as described previously (Mccausland and Crotty, 2008).

Statistical analysis

Statistical analysis was conducted with Prism 6.0 (GraphPad). Paired or unpaired two-tailed *t*-test with 95% confidence interval was used for calculation of *P*-values.

Supplementary Figure S1

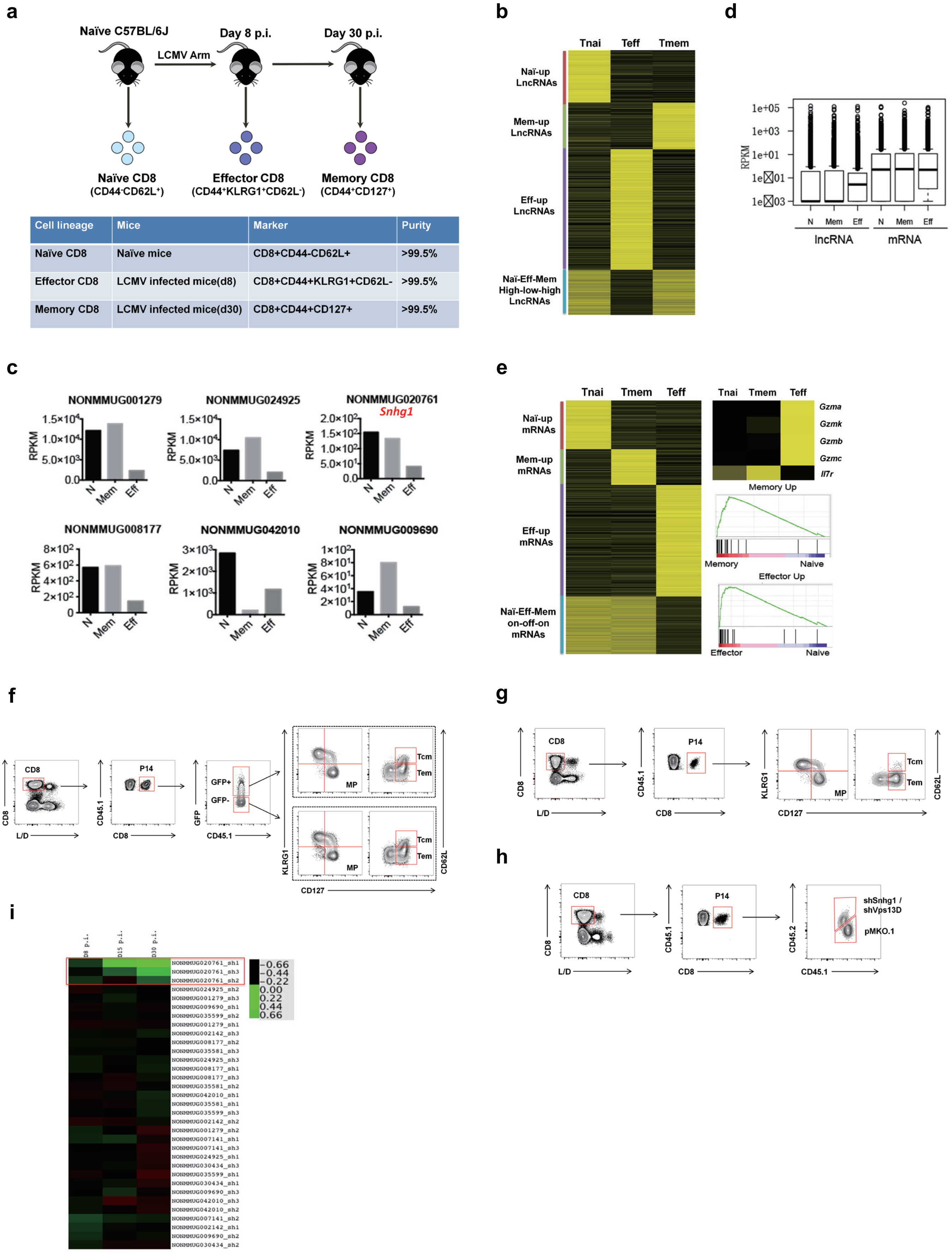

Cell lineage	Mice	Marker	Purity
Naïve CD8	Naïve mice	CD8+CD44-CD62L+	>99.5%
Effector CD8	LCMV infected mice(d8)	CD8+CD44+KLRG1+CD62L-	>99.5%
Memory CD8	LCMV infected mice(d30)	CD8+CD44+CD127+	>99.5%

Figure S1. LncRNA Snhg1 from CD8 T cell subsets profiling is required for memory CD8 T cell differentiation. Related to Figure 1.

(**a**) Naïve, effector and memory CD8 T cells with the indicated cell markers were sorted from LCMV Armstrong virus-infected mice on indicated days for RNA-seq analysis. Purity of the indicated cells were listed at the bottom. (**b**) Heatmap of lncRNAs that were clustered with expression level in naïve, effector and memory CD8 T cells according to the fold-change with cutoff of 2. (**c**) The RPKMs of the top candidate lncRNAs in naïve, effector and memory CD8 T cells (red marked Snhg1). (**d**) The total RPKM of protein-coding genes (mRNAs) and long-noncoding genes (lncRNAs) in naïve, effector and memory CD8 T cells. (**e**) Heatmap of mRNAs that were clustered with expression level in naïve, effector and memory CD8 T cells according to the fold-change with cutoff of 2 (left). The right were GSEA analysis of genes upregulated in memory or effector CD8 T cells with showing the representative effector-up *Granzyme* gene isoforms and the memory-up gene *Il7r*. (**f-h**) The gating strategy for Fig 1d-k. (**f**) is for Fig. 1d, 1g and 1h; (**g**) is for Fig. 1e, 1f and 1k, and (**h**) is for Fig. 1i. (**i**) log2 ratio of $CD127^{+}KLRG1^{-}$ % in GFP^{-} to that of GFP^{+} P14 cells by using shRNAs to target the candidate lncRNAs for internal analysis on days 8, 15 and 30 p.i.. Data are representative of two or three independent experiments with at least three replicates or four mice per group.

Supplementary Figure S2

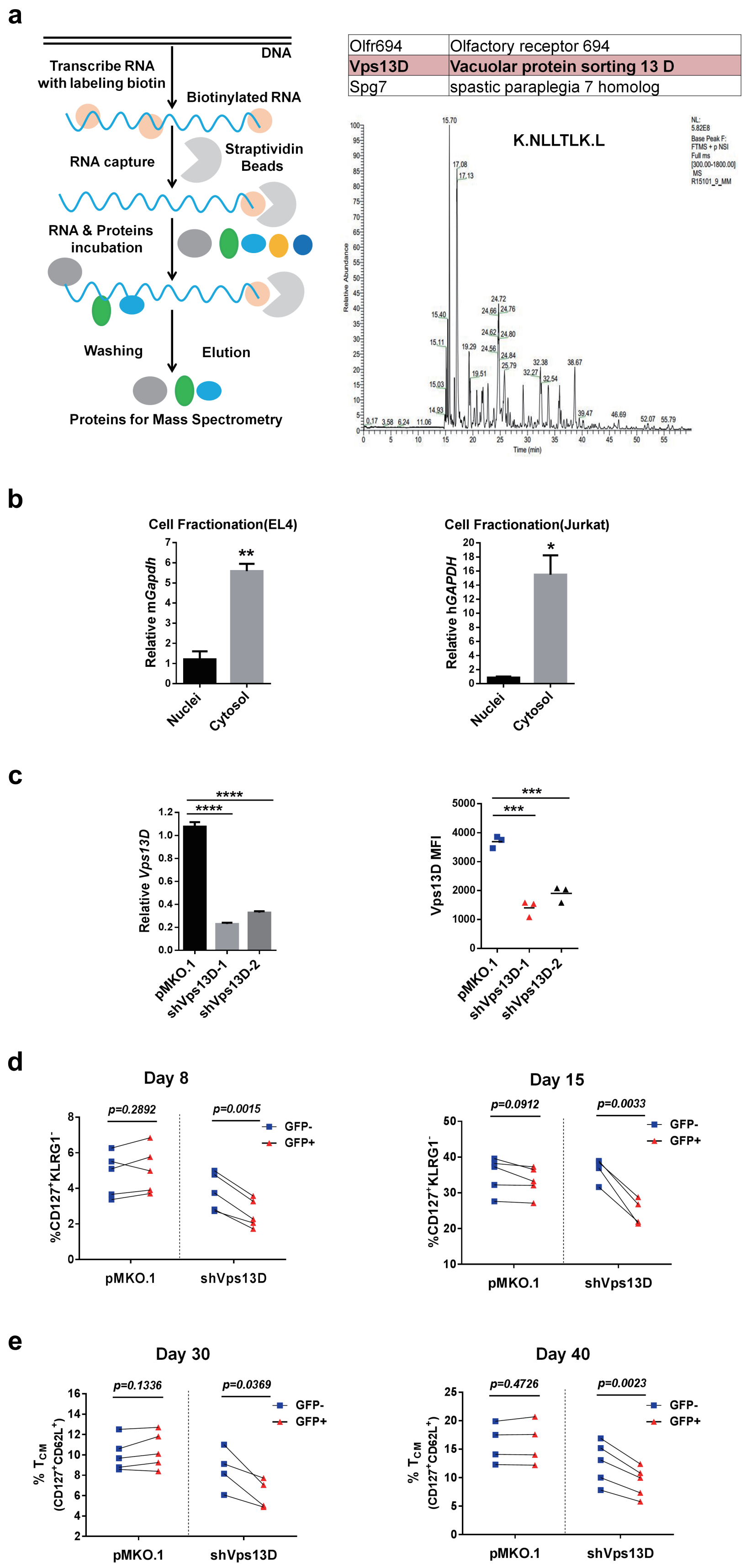

Figure S2. RNA pulldown coupled with MS uncovers the Snhg1 interacted protein Vps13D that is required for memory CD8 T cell establishment. Related to Figure 2.

(**a**) A sketch of Snhg1 pulldown experiment in sorted memory CD8^{+} T cells (left) and the identified proteins by mass spectrometry showing the protein of interest, Vps13D. (**b**) RT-qPCR of *Gapdh* (*GAPDH*) in separated nuclei or cytosol from El4 (or Jurkat) cells. (**c**) RT-qPCR of *Vps13D* and mean fluorescent intensity (MFI) of Vps13D protein in memory CD8 T cells with the two shRNA sequences of *Vps13D* transducted. (**d, e**) The internal analysis of CD127^{+}KLRG1^{-} population (**d**) and central memory CD8 T cells (**e**) by comparing the GFP^{-} with GFP^{+} P14 CD8 T cells in the same mice in control or sh*Vps13D* groups on indicated days (experiment of Fig. S2d right was done side by side with that of Fig. 1h). Data are representative of two or three independent experiments with at least three replicates or four mice per group (error bars denote s.e.m.). **p* < 0.05, ***p* < 0.01, *****p* < 0.0001 (paired or unpaired two-tailed *t*-test).

Supplementary Figure S3

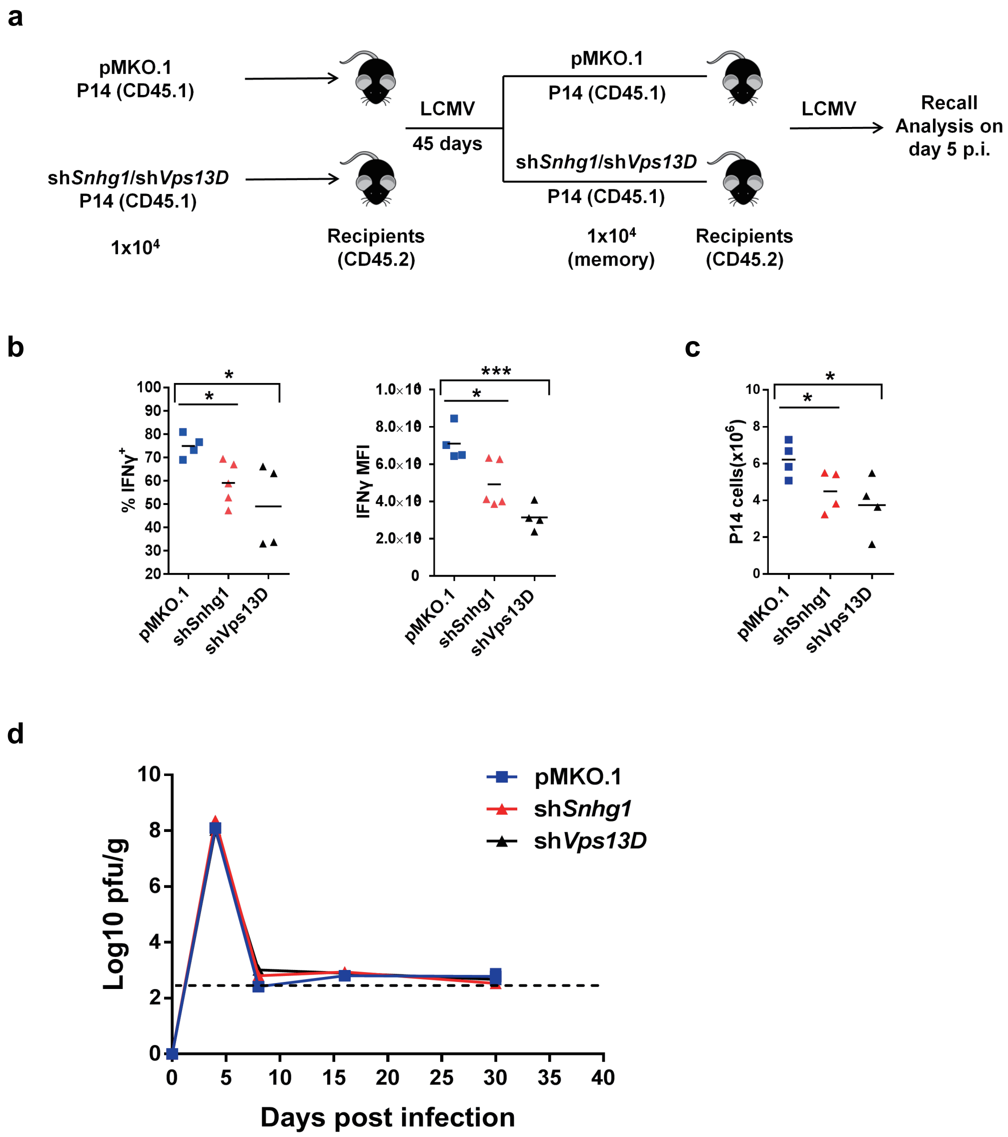

Figure S3. Snhg1 and Vps13D preserve the function of memory CD8 T cells. Related to Figure 3.
(**a-c**) The scheme of recall experiment (**a**). The proportion and MFI of IFN γ^+ cells (**b**) and the cell number of P14 cells on day 5 post re-infection (**c**). (**d**) The sorted 2000 GFP$^+$ P14 cells (CD45.1) transducted with retrovirus expressing pMKO.1/shSnhg1/shVps13D were injected into CD45.2 mice followed with LCMV infection, the kinetics of viral titers in spleen with infection timeline were shown. Data are representative of two or three independent experiments with at least four mice per group (error bars denote s.e.m.). ns, not significant; *$p < 0.05$, **$p < 0.01$, ***$p < 0.001$ (unpaired two-tailed t-test).

Supplementary Figure S4

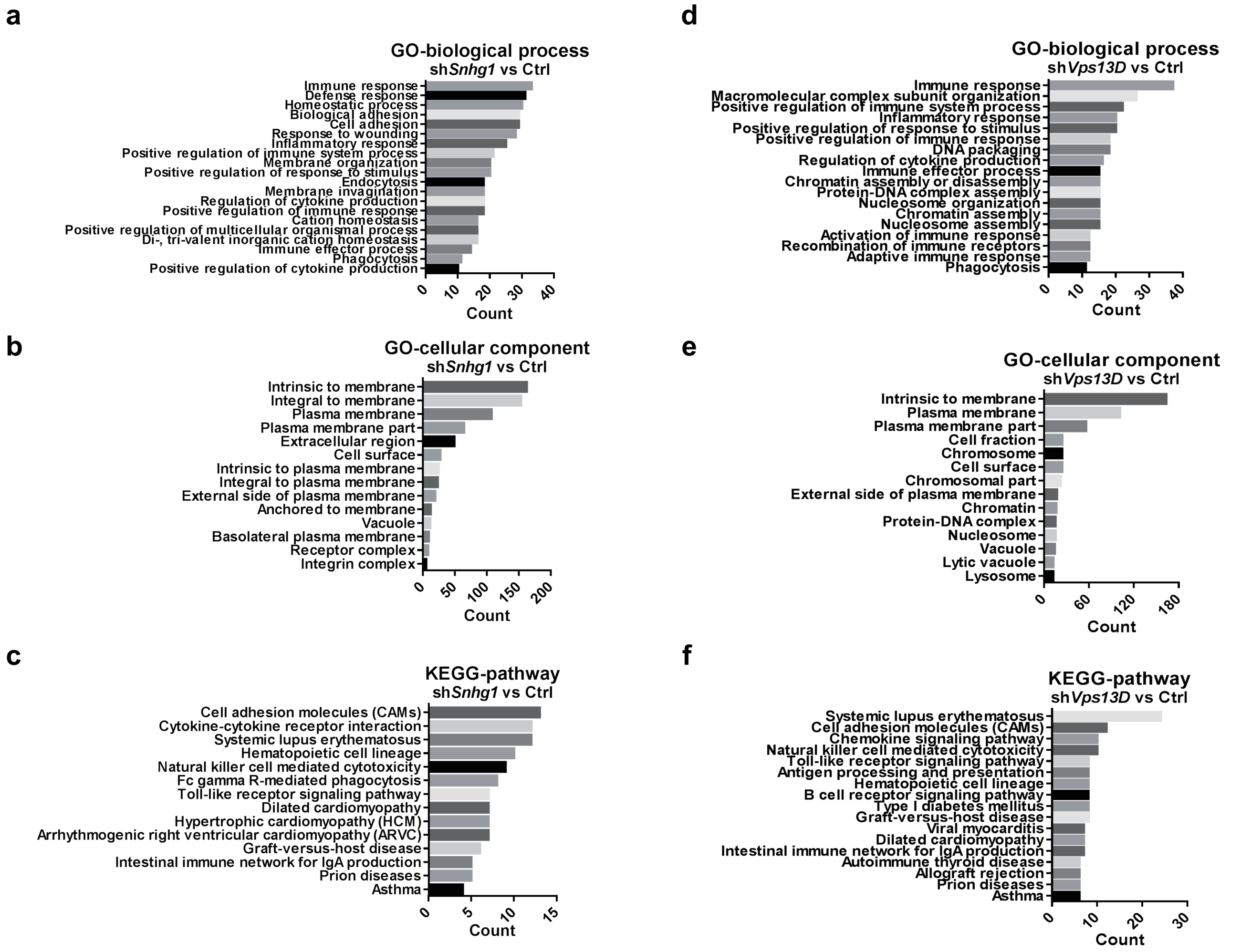

Figure S4. Genes modulated by Snhg1 or Vps13D in T cells mainly involve in membrane receptor-associated immune processes. **Related to Figure 4.**

(**a-f**) GO and KEGG analysis of regulated genes by sh*Snhg1* or sh*Vps13D* in biological processes, cellular components and pathways compaired with the control pMKO.1 group on day 17 post infection in the transition phase. Data are obtained from one experiment with two biological replicates pooled from at leat four mice per group.

Supplementary Figure S5

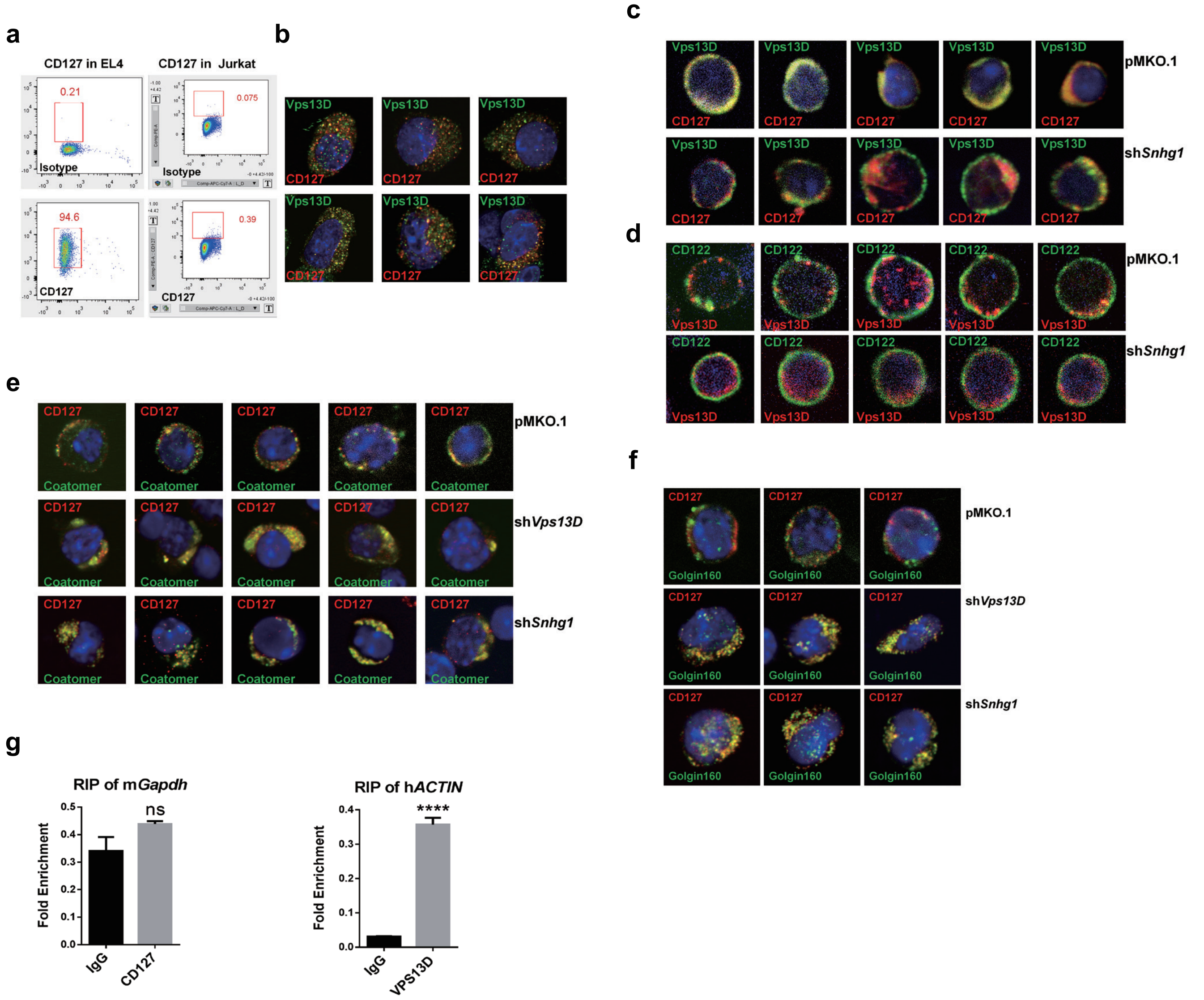

Figure S5. Snhg1 and Vps13D specifically regulate the trafficking of IL-7Rα from ER-Golgi to cell membrane. Related to Figure 5.

(a) Flow cytometry analysis of CD127 expression in El4 cells or Jurkat cells. (**b**) Confocal microscopy of CD127 (red) and Vps13D (green) in El4 cells. (**c, d**) Confocal microscopy of CD127 (**c**) or CD122 (**d**) with Vps13D in the sorted sh*Snhg1*/pMKO.1 GFP^{+} P14 cells on day 35 post infection. (**e, f**) Confocal microscopy of CD127 (red) with Coatomer (**e**) or Golgin160 (**f**) (green) in the sorted sh*Snhg1*/sh*Vps13D*/pMKO.1 GFP^{+} P14 cells on day 20 post infection. (**g**) RIP assay of m*Gapdh* using rabbit anti-CD127 in El4 cells (left) and RIP assay of h*ACTIN* using rabbit anti-Vps13D in Jurkat cells (right) compared with the normal Rabbit IgG control. Data are representative of two or three independent experiments with at least four mice per group (paired two-tailed *t*-test).

Supplementary Figure S6

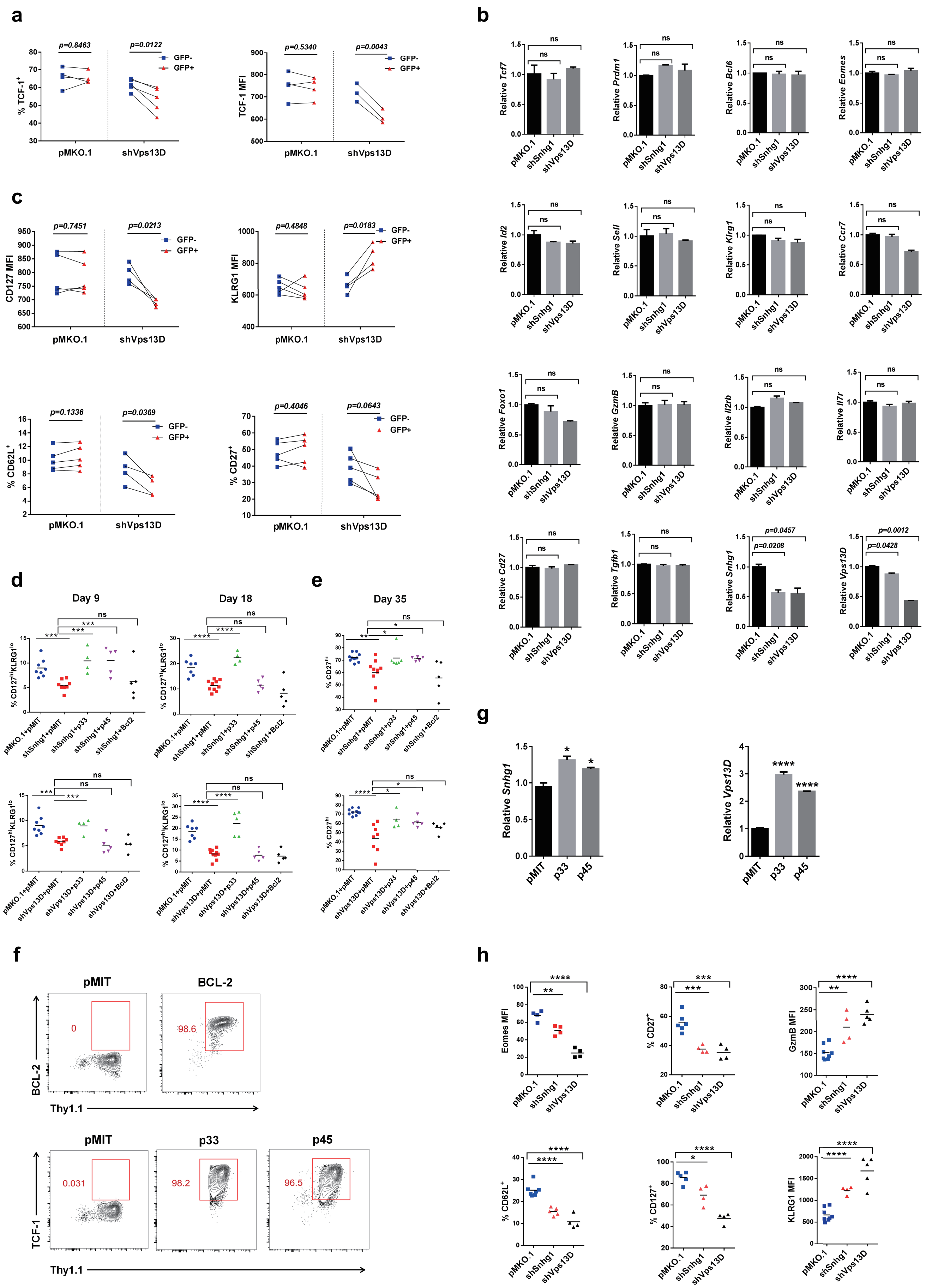

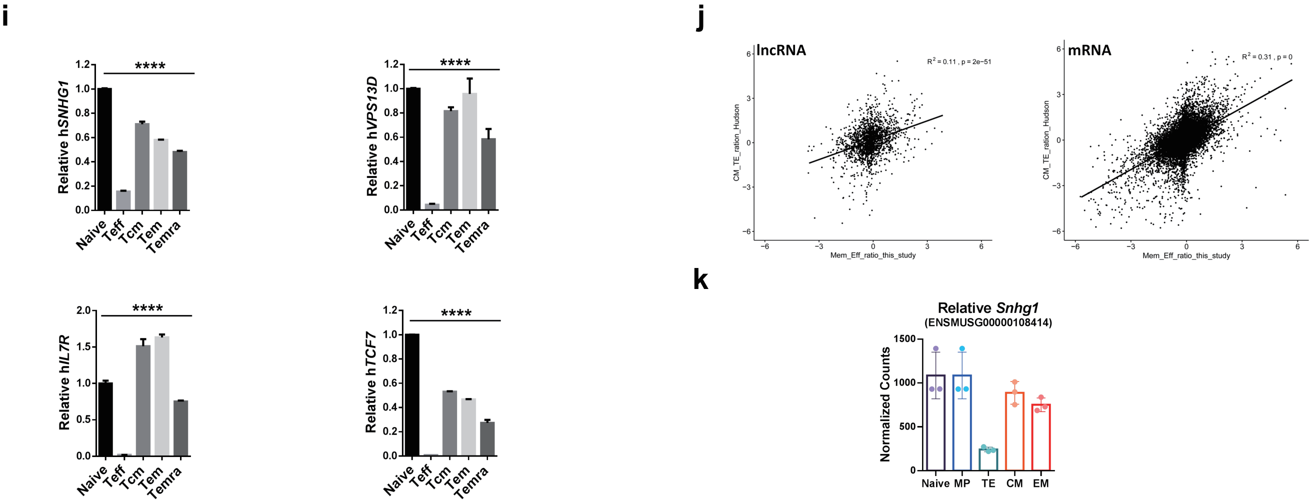

Figure S6. Snhg1 and Vps13D depletion do not affect the transcriptome in memory maintenance stage. Related to Figure 6.
(**a**) The internal analysis of the proportion of TCF-1^{+} cells and TCF-1 MFI by comparing the GFP^{-} with GFP^{+} P14 CD8 T cells in the same mice in control or sh*Vps13D* groups on day 35 p.i.. (**b**) The established GFP^{+} P14 CD8 T cells were sorted for RT-qPCR analysis of genes on day 45 p.i.. (**c**) The internal analysis of proteins by comparing the GFP^{-} with GFP^{+} P14 CD8 T cells in the same mice in control or sh*Vps13D* groups on day 35 p.i.. (**d, e**) FACS analysis of CD127^{hi}KLRG1^{lo} and CD27^{hi} cells in double-positive (GFP^{+}Thy1.1^{+}) P14 cells on indicated days p.i.. (**f**) Flow cytometry of BCL-2 and TCF-1 (p33 and p45) in 293T cells that were transfected with pMIT/*Tcf7*/*Bcl2* (IRES-*Thy1.1*) for 24 hrs. (**g**) The established Thy1.1^{+} P14 cells were sorted for RT-qPCR of *Snhg1*/*Vps13D* on day 50 p.i. (**h**) Flow cytometry analysis of the indicated proteins in pMKO.1/sh*Snhg1*/sh*Vps13D* GFP^{+} P14 CD8 T cells on day 35 p.i. (**i**) RT-qPCR analysis of human *SHHG1*/*VPS13D*/*IL7R*/*TCF7* in CD8 T cell lineages from PBMC of the convalescent COVID-19 patients. (**j**) Scatter plot to show the ratio of lncRNAs expression between memory cells and effector cells of Hudson *et al* along y axis, and that of this study along x axis (Correlation coefficient was calculated with Pearson correlation). (**k**) Relative *Snhg1* expression level with normalized counts in naïve, effector, MP, Tcm and Tem cells analized from RNA-seq data of Hudson *et al*. Data are representative of two or three independent experiments with at least three replicates or four mice per group (error bars denote s.e.m.). ns, not significant; *$p < 0.05$, **$p < 0.01$, ***$p < 0.001$, ****$p < 0.0001$(paired or unpaired two-tailed *t*-test).

Supplementary Table S1

Table S1. Antibodies and reagents used in flow cytometry. Related to Figure 1-6.

**Antibody/Reagent**	**Cat.#**	**Provider**
CD127	A7R34	Biolegend
CD8	53-6.7	Biolegend
KLRG1	138408	Biolegend
CD25	PC61.5	Biolegend
CD44	IM7	Biolegend
CD45.1	A20	Biolegend
CD122	TM-β1	Biolegend
CD62L	MEL-14	Biolegend
CD132	TUGm2	Biolegend
CD90.1	OX-7	Biolegend
TCF-1	C46C7	Cell Signalling Technology
BCL-2	556537	BD Biosciences
Annexin V kit	559763	BD Biosciences
p-STAT3 (Tyr705)	D3A7	Cell Signalling Technology
p-STAT5 (Tyr694)	C71E5	Cell Signalling Technology
BCL-6	7D1	BioLegend
Ki-67	556027	BD Biosciences
CD27	LG.3A10	BioLegend
anti-rabbit IgG A647	4414S	Cell Signalling Technology
Eomes	Dan11mag	Thermo Fisher Scientific
TNF-α	MP6-XT22	BD Biosciences
CD107a	50-1071-80	Thermo Fisher Scientific
CD107b	50-1072-82	Thermo Fisher Scientific
p-AKT(S473)	D9E	Cell Signalling Technology
Vα2	B20.1	BioLegend
IFN-γ	XMG1.2	BioLegend
Granzyme B	GB11	Thermo Fisher Scientific
IL-2	JES6-5H4	BioLegend
hCD3	HIT3a	BD Biosciences
hCD8	HIT8a	BD Biosciences
hCCR7	3D12	BD Biosciences
hCD45RA	HI100	BD Biosciences

Supplementary Table S2

Table S2. Top lncRNA candidates from lncRNAseq of naïve/effector/memory CD8 T cells. Related to Figure 1.

lncRNA	chr	start	end	Length of exonclass		Naive	Effector	Memory
NONMMUG001279	chr1	89673513	89673784	271,	Sense	12282.8	2354.36	14073.8
NONMMUG024925	chr2	144091715	144091952	237,	Sense	7496.85	2080.31	10668.8
NONMMUG020761	chr19	8797897	8800935	3038,	Linc,	156.194	41.0437	135.407
NONMMUG008177	chr11	116938097	116940269	2172,	Linc,	577.088	149.216	602.817
NONMMUG007141	chr11	86625330	86629631	4301,	Linc,	476.906	219.757	291.15
NONMMUG042010	chr9	3000282	3168181	348,...,335,	Linc,	2902.46	1177.04	200.811
NONMMUG009690	chr12	94121102	94123634	97,124,934,91,	Linc,	35.8955	12.2219	81.5867

*Gene highlighted in yellow is validated Snhg1 by RT-qPCR.

Supplementary Table S3

Table S3. Primers and shRNA sequences. Related to Figure 1-6.

**3.1 Primers used in gene expression or RIP analysis. Related to Figure 1-2, 5-6.**		
**Gene symbol**	**Forward primer**	**Reverse primer**
Ki67	CCAGCTGCCTGTAGTGTCAA	CCATGTCTCAGCCTCACAGG
Bcl2	GGGGTCATGTGTGTGGAGAG	ACCTACCCAGCCTCCGTTAT
Cd107	AGCATACCGGTGTGTCAGTG	GTTGGGGAAGGTCCATCCTG
Perforin1	CTTGTGACAGGTCTCCCCAC	AGGAGAATTTCACCCTGCCG
Vps13D	GATGGTGGTAGTGGTGTCCG	GACCTCGGCCTTTCTTGACA
Snhg1	TCCTTGTTCGGGGTTTGAGG	ACAGCACCCTGACTACAAGC
Prdm1	AACGTGTGGGTACGACCTTG	TTCAAACTCGGCCTCTGTCC
Bcl6	CTGCACTTAAACCTCCCCGT	TTGTTCTCCACGACCTCACG
Ccr7	TTGCCGTGGTGGTAGTCTTC	GCAATGTTGAGCTGCTTGCT
Cd27	GTGTGAGCCAGAGAAGCCAT	GTCTATGGGATGACCGCTGG
Eomes	TCCGGGACAACTACGATTCAT	CGGGAAGAAGTTTTGAACGC
Tbx21	TGTTCCCAGCCGTTTCTACC	GCTCGGAACTCCGCTTCATA
GzmB	ACAACACTCTTGACGCTGGG	CGAGAGTGGGGCTTGACTTC
Tcf7	GTAAACAGACCCCCGCCATC	GGGTAGGGCATGAGCAGATT
p45	GGCGAGGAACAGGACGATAA	AGTGAGGACTTGAGCTCTGC
Il2ra	ACCTGGCAACACAGATGGAG	TTGGCGTCTCAGATTTGGCT
Id2	AAAGCCTTCAGTCCGGTGAG	GAGCTTGGAGTAGCAGTCGT
Id3	GCCCGAGAGAAGGACTGAAC	CGACACCCCATTCTCGGAAA
Tgfb1	CTGCTGACCCCCACTGATAC	AGCCCTGTATTCCGTCTCCT
Foxo1	AGTGGATGGTGAAGAGCGTG	GAAGGGACAGATTGTGGCGA
Sell	CTGGCATTTCTCATTTGGCT	TTCATGGCTTTCCTTTCACA
Il2rb	GGTCTCCCAGGTCTCTCACT	CCACTGCTGTCTCTGCTTGA
Il7r	TCGTATGGCCTAGTCTCCCC	CACATTCAGACTCGTTTTTGGCT
Klrg1	TTTGGGGCTTTTGACTGTGAT	TGTAAGGAGATGTGAGCCTTTGT
Ifng	TATCTGGAGGAACTGGCAAA	GGTGTGATTCAATGACGCTT
Tnfa	CCCTCACACTCACAAACCAC	AGGGTTTGCTACAACATGGG
Il2	AAGCAGGCCACAGAATTGA	TGAAATTCTCAGCATCTTCCAA
Gapdh	GGCAAATTCAACGGCACAGT	GTCTCGCTCCTGGAAGATGG
Hprt	CTTCCTCCTCAGACCGCTTT	TCATCGCTAATCACGACGCT
**h*IL7R***	TCTCTGTCGCTCTGTTGGTC	CTGGGCCATACGATAGGCTT
**h*SNHG1***	TGTTGCAATGTTCAGCCCAC	GTATTCACCCTGGGAGGCAG
**h*VPS13D***	TTGGACAAACAGAGGTGAAAACC	CAGGGTGGCAAACCACAAAG

h*GAPDH*	GGCAAATTCCATGGCACCG	AGCATCGCCCCACTTGATTT
**3.2 Primers used in ChIP analysis. Related to Figure 6.**		
**Gene location**	**Forward primer**	**Reverse primer**
Tcf7 -31.8k	ATAAACACTGTGGGCCAAGG	CCACGAGATAGGGCAACAAT
Tcf7 -17.5k	CAGGGTGTTTTAGCGGAGAG	CAGCAACAGACACAGCCAGT
Prdm1 -24.5k	ATGTTTCGGAGGAGGTTGTG	AAGGGTGGGACCAGATTCTT
Prdm1 +0.9k	TCAGCCCCAGTCTGTCTTCT	GGCAAAAGACCATTGAAGGA
Il7r -3.9k	TTGTGGGTGAGCAATGGTAA	TCTGACAAAGCAGTGCCATC
Il7r +2.4k	GCCACCCAGTCTGACAAAGT	GTGCCCCAGTAGCAACATCT
Bcl6 -0.5k	GGGTCTGGGGCTAATTCTTC	TAGCTGGAAGGAGCTGTGGT
Bcl6 +2.9k	GGCTCAACCCATAACTGGAA	TTATTCTGGCCTTGGTGGTC

**3.3 shRNA used in retrovirus transduction. Related to Figure 1-6.**	
**sh*Snhg1*-F1**	CCGGAAAGTTGAAGCATATCTTCATCTCGAGATGAAGATATGCTTCAACTTTTTTTG
**sh*Snhg1*-R1**	AATTCAAAAAAAAGTTGAAGCATATCTTCATCTCGAGATGAAGATATGCTTCAACTTT
**sh*Snhg1*-F2**	CCGGAATCATGAAAGATCTGTTTGTCTCGAGACAAACAGATCTTTCATGATTTTTTG
**sh*Snhg1*-R2**	AATTCAAAAAAATCATGAAAGATCTGTTTGTCTCGAGACAAACAGATCTTTCATGATT
**sh*Vps13D*-F1**	CCGGATGTCGTCAGCATCGGAAATTCTCGAGAATTTCCGATGCTGACGACATTTTTG
**sh*Vps13D*-R1**	AATTCAAAAATGTCGTCAGCATCGGAAATTCTCGAGAATTTCCGATGCTGACGACAT
**sh*Vps13D*-F2**	CCGGGACAAAGCCTCCCTCTTGTCTCTCGAGAGACAAGAGGGAGGCTTTGTCTTTTTG
**sh*Vps13D*-R2**	AATTCAAAAAGACAAAGCCTCCCTCTTGTCTCTCGAGAGACAAGAGGGAGGCTTTGTC

Supplementary Table S4

Table S4. Protein hits enriched from Snhg1 pulldown coupled mass spectrometry. Related to Figure 2.

ID	FileScan	Rank
$1126-2	tr|B1ART1|B1ART1_MOUSE Protein Vps13d OS=Mus musculus GN=Vps13d PE=1 SV=1	6.07
$1126-3	tr|Q6A066|Q6A066_MOUSE MKIAA0453 protein (Fragment) OS=Mus musculus GN=Vps13d PE=2 SV=1	6.68
$1126-1	tr|V9GX23|V9GX23_MOUSE Protein Vps13d (Fragment) OS=Mus musculus GN=Vps13d PE=1 SV=1	6.38
$1126-4	tr|B1ART2|B1ART2_MOUSE Protein Vps13d OS=Mus musculus GN=Vps13d PE=1 SV=2	6.11
$1046-1	tr|Q8VF88|Q8VF88_MOUSE Olfactory receptor 694 OS=Mus musculus GN=Olfr694 PE=2 SV=1	7.52
$1046-3	tr|K7N641|K7N641_MOUSE Protein Olfr694 OS=Mus musculus GN=Olfr694 PE=4 SV=1	7.52
$970-1	tr|Q7TNG0|Q7TNG0_MOUSE Spg7 protein (Fragment) OS=Mus musculus GN=Spg7 PE=2 SV=1	8.87
$970-4	tr|F6W695|F6W695_MOUSE Paraplegin (Fragment) OS=Mus musculus GN=Spg7 PE=1 SV=1	7.31
$970-7	tr|D3YZN4|D3YZN4_MOUSE Paraplegin OS=Mus musculus GN=Spg7 PE=1 SV=1	6.96
$970-2	tr|D3YXB7|D3YXB7_MOUSE Paraplegin OS=Mus musculus GN=Spg7 PE=1 SV=1	9.3
$970-3	tr|B2RQY8|B2RQY8_MOUSE Spastic paraplegia 7 homolog (Human) OS=Mus musculus GN=Spg7 PE=2 SV=1	9.09
$970-5	sp|Q3ULF4|SPG7_MOUSE Paraplegin OS=Mus musculus GN=Spg7 PE=1 SV=1	9.09

$970-6	tr|D3Z1Z1|D3Z1Z1_MOUSE Paraplegin OS=Mus musculus GN=Spg7 PE=1 SV=1	9.09
$970-8	tr|F6VTG4|F6VTG4_MOUSE Paraplegin (Fragment) OS=Mus musculus GN=Spg7 PE=1 SV=1	8.04

Supplementary Table S5

Table S5. Representative genes in mRNAseq of sh*Snhg1*/sh*Vps13D*/pMKO.1 transducted memory CD8 T cells. Related to Figure 4.

Gene	pMKO.1	sh*Vps13D*	sh*Snhg1*
Il7r	103.712	64.9831	77.6771
Klrg1	120.821	209.171	157.962
Sell	16.6125	11.4821	7.04716
Prdm16	0.089396	0.150115	0.563287
Prdm11	5.63477	7.18088	5.99988
Prdm15	5.91006	6.13944	9.39462
Prdm1	2.02602	4.26322	5.10455
Prdm10	3.58466	4.13485	4.73308
Tcf7	15.7274	10.2556	10.1536
Tcf7l2	0.399192	0.190925	0.405545
Eomes	7.97616	4.13421	6.02046
Bcl6	2.12786	0.969399	1.56253
Ccr7	3.65116	1.13288	2.35251
Ccr9	1.2993	0.77647	1.29695
Ccr1	0.325351	0.542776	0.162097
Ccrl1	0.207038	0.141131	0.142176
Ccr3	9.76723	2.58395	1.85771
Ccr6	0.194948	0.070946	0.104145
Bcl2	18.6909	15.6953	15.1514
Bcl2a1b	49.5123	39.7411	39.9611
Bcl2a1a	7.98624	6.46873	6.09322
Il2ra	8.108	5.57053	5.78041
Il2rb	128.478	128.407	146.422
Il2rg	55.3834	56.6989	53.6196
Stat2	3.94694	4.66186	4.54334
Stat4	75.6078	63.5783	76.8251
Stat6	51.1964	62.2191	64.9349
Stat1	39.9107	46.5789	39.8084
Stat3	48.574	43.8277	49.1942
Stat5b	18.0846	20.4253	24.6027
Stat5a	12.8251	12.9632	16.0389
Gzmb	169.816	277.643	255.2
Ifng	89.7901	70.4257	80.339
Ifnk	0.06135	0.128269	0.233116
Ifngr2	0.482592	0.108857	0.212258
Ifngr1	95.0464	84.9765	105.682
Ifnar2	40.4987	37.4893	39.9108

Supplementary Table S6

Table S6. Protein hits enriched from Vps13D pulldown coupled mass spectrometry. Related to Figure 5.

tr|Q8BTF0|Q8BTF0_MOUSE Coatomer subunit alpha OS=Mus musculus OX=10090 GN=Copa PE=2 SV=1
sp|Q8CIE6|COPA_MOUSE Coatomer subunit alpha OS=Mus musculus OX=10090 GN=Copa PE=1 SV=2
sp|Q5XJY5|COPD_MOUSE Coatomer subunit delta OS=Mus musculus OX=10090 GN=Arcn1 PE=1 SV=2
tr|Q8C0G7|Q8C0G7_MOUSE Coatomer subunit delta OS=Mus musculus OX=10090 GN=Arcn1 PE=2 SV=1
sp|Q9QZE5|COPG1_MOUSE Coatomer subunit gamma-1 OS=Mus musculus OX=10090 GN=Copg1 PE=1 SV=1
sp|P61750|ARF4_MOUSE ADP-ribosylation factor 4 OS=Mus musculus OX=10090 GN=Arf4 PE=1 SV=2
sp|P61205|ARF3_MOUSE ADP-ribosylation factor 3 OS=Mus musculus OX=10090 GN=Arf3 PE=2 SV=2
sp|P84078|ARF1_MOUSE ADP-ribosylation factor 1 OS=Mus musculus OX=10090 GN=Arf1 PE=1 SV=2
sp|Q8BSL7|ARF2_MOUSE ADP-ribosylation factor 2 OS=Mus musculus OX=10090 GN=Arf2 PE=1 SV=2
tr|Q3U344|Q3U344_MOUSE ADP-ribosylation factor 3 OS=Mus musculus OX=10090 GN=Arf3 PE=2 SV=1
sp|P62331|ARF6_MOUSE ADP-ribosylation factor 6 OS=Mus musculus OX=10090 GN=Arf6 PE=1 SV=2
tr|A0A0R4J1H6|A0A0R4J1H6_MOUSE Golgin subfamily A member 3 OS=Mus musculus OX=10090 GN=Golga3 PE=1 SV=1
sp|P55937|GOGA3_MOUSE Golgin subfamily A member 3 OS=Mus musculus OX=10090 GN=Golga3 PE=1 SV=3

tr|E9QP99|E9QP99_MOUSE Golgin subfamily A member 3 OS=Mus musculus OX=10090 GN=Golga3 PE=1 SV=1
sp|P61620|S61A1_MOUSE Protein transport protein Sec61 subunit alpha isoform 1 OS=Mus musculus OX=10090 GN=Sec61a1 PE=1 SV=2
tr|Q3TJD0|Q3TJD0_MOUSE Uncharacterized protein OS=Mus musculus OX=10090 GN=Sec61a1 PE=2 SV=1
sp|P61294|RAB6B_MOUSE Ras-related protein Rab-6B OS=Mus musculus OX=10090 GN=Rab6b PE=1 SV=1
tr|Q0PD53|Q0PD53_MOUSE RAB6B, member RAS oncogene family OS=Mus musculus OX=10090 GN=Rab6b PE=1 SV=1
